# Nanomedicine Meets Immunotherapy: Transforming Chimeric Antigen Receptor T Cell Treatment for Solid Tumors

**DOI:** 10.1002/smsc.202500596

**Published:** 2026-02-02

**Authors:** Stephen O’Rourke, Nuo Xu, Hongjun Wang, Hexin Chen

**Affiliations:** ^1^ Department of Biological Sciences University of South Carolina Columbia SC 29208 USA; ^2^ Department of Biomedical Engineering Stevens Institute of Technology Hoboken NJ 07030 USA

**Keywords:** chimeric antigen receptor T cell therapy, nanomedicine, solid tumor, targeted delivery, tumor microenvironment

## Abstract

The emergence of effective immunotherapies has drastically revolutionized clinical management of many cancer types. Among them, chimeric antigen receptor (CAR)‐T cell therapy (CTT), as a groundbreaking approach, has been considered as a “living drug,” displaying unprecedented clinical outcomes with hematological malignancies, including B cell leukemia and lymphomas, and multiple myeloma. Despite the high remission rates and improved survival achieved with hematological cancers, the effectiveness of CTT in solid tumors remains largely unsatisfactory. The efficacy of CTT in solid tumors is significantly challenged by multiple factors, including tumor‐antigen heterogeneity, limited T cell trafficking and infiltration, a highly immunosuppressive tumor microenvironment, and the risk of severe adverse effects. Accumulating evidence highlights the potential of nanotechnology to address these obstacles, paving the way for more effective CTT against solid tumors. Thus, this review explores to highlight the evolution and challenges of CTT in solid tumors, while summarizing the up‐to‐date advances of nanotechnology‐enabled CTT with the intention towards the formulation of a more cohesive, personalized, and effective cancer therapy in the future.

## Graphical abstract

This review highlights recent advances in nanomedicine that enhance chimeric antigen receptor (CAR) T cell therapy against solid tumors. It discusses key barriers to CAR T cell efficacy and outlines nanotechnology‐based strategies to improve tumor targeting, remodel the immunosuppressive microenvironment, and integrate nanomaterials into CAR T cell platforms for improved therapeutic outcomes.

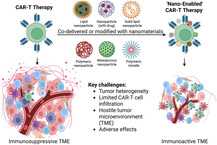

## 1 Introduction

Immunotherapy has held revolutionary promise to treat cancers, particularly those metastatic ones deemed incurable, with the potentials to achieve long‐term remission and possible cure.^[^ ^1^ ^]^ For instance, immune checkpoint inhibitor (ICI)‐targeted immunotherapy has greatly ameliorated the overall survival of patients with cancers such as melanoma and nonsmall cell lung cancer.^[^ ^1^, ^2^, ^3^, ^4^, ^5^, ^6^ ^]^ However, abundant evidence also show that a large population of patients do not respond to ICI treatments or suffer from the acute clinical toxicities of these ICI treatments.^[^ ^7^, ^8^, ^9^, ^10^ ^]^ Endeavors have also been extended to combine immunotherapy with conventional treatments such as surgery, radiation, chemotherapy to treat those aggressive tumors. However, such combination therapies often show the risk of increasing toxicity. Thus, other robust immunotherapeutic strategies are highly desirable in order to improve the efficacy, reduce the unwanted toxicity, and expand the spectrum of beneficial outcomes, especially for the patients with metastatic cancers.

Adoptive cell therapy (ACT), pioneered by *Rosenberg*, is a promising immunotherapy involving the extraction, activation, and reinfusion of patients’ T cells to enhance the ability of their immune system to combat cancers.^[^ ^11^, ^12^, ^13^, ^14^, ^15^ ^]^ ACT encompasses three representative mainstreams: tumor‐infiltrating lymphocytes (TILs), T cell receptor‐engineered T cells (TCR‐T), and chimeric antigen receptor (CAR) modified T cells.^[^ ^16^ ^]^ Early studies with TILs indeed demonstrated the antitumor activity of lymphocytes isolated from patient‐derived tumors, particularly those of melanoma.^[^ ^17^ ^]^ However, isolation and expansion of the tumor‐reactive TILs from solid tumors is very challenging, and such TILs oftentimes lose their capability of effectively recognizing tumor antigens.^[^ ^18^ ^]^ As such, efforts have also been geared towards the introduction of genetically engineered tumor‐specific T cell receptors (TCRs) into autologous lymphocytes. Both conventional αβ TCRs (TCR‐T) or CAR T cells with antitumor specificity can be introduced into normal lymphocytes, endowing them with antitumor activity and systemic immune responses.^[^ ^18^, ^19^ ^]^

For TCR‐T, tumor‐specific TCRs can be obtained from patients’ peripheral blood mononuclear cells (PBMCs), or TILs, or derived from tumor antigen‐immunized HLA‐I/II transgenic mice^[^ ^20^, ^21^ ^]^ and then introduced into patients’ peripheral blood T cells via retroviral or lentiviral vectors for expression. TCRs derived from HLA‐I/II transgenic mice generally exhibit higher affinity than those derived from PBMCs or TILs. TCR‐transduced T cells specific for tumor antigens such as MART1, CEA, gp100, NY‐ESO‐1, and MAGEA3 have been evaluated in clinical trials.^[^ ^22^, ^23^, ^24^ ^]^ While exhibiting significantly enhanced ability to treat various cancers, these TCR‐T therapeutic strategies also face noted limitations. One of the key challenges in TCR‐based immunotherapy is the inherently low binding affinity of natural TCRs to tumor antigens. Conversely, artificial increase in TCR affinity can lead to severe adverse events due to the cross reactivity with low‐level antigens in the normal tissues.^[^ ^20^, ^25^, ^26^ ^]^

**Figure 1:**
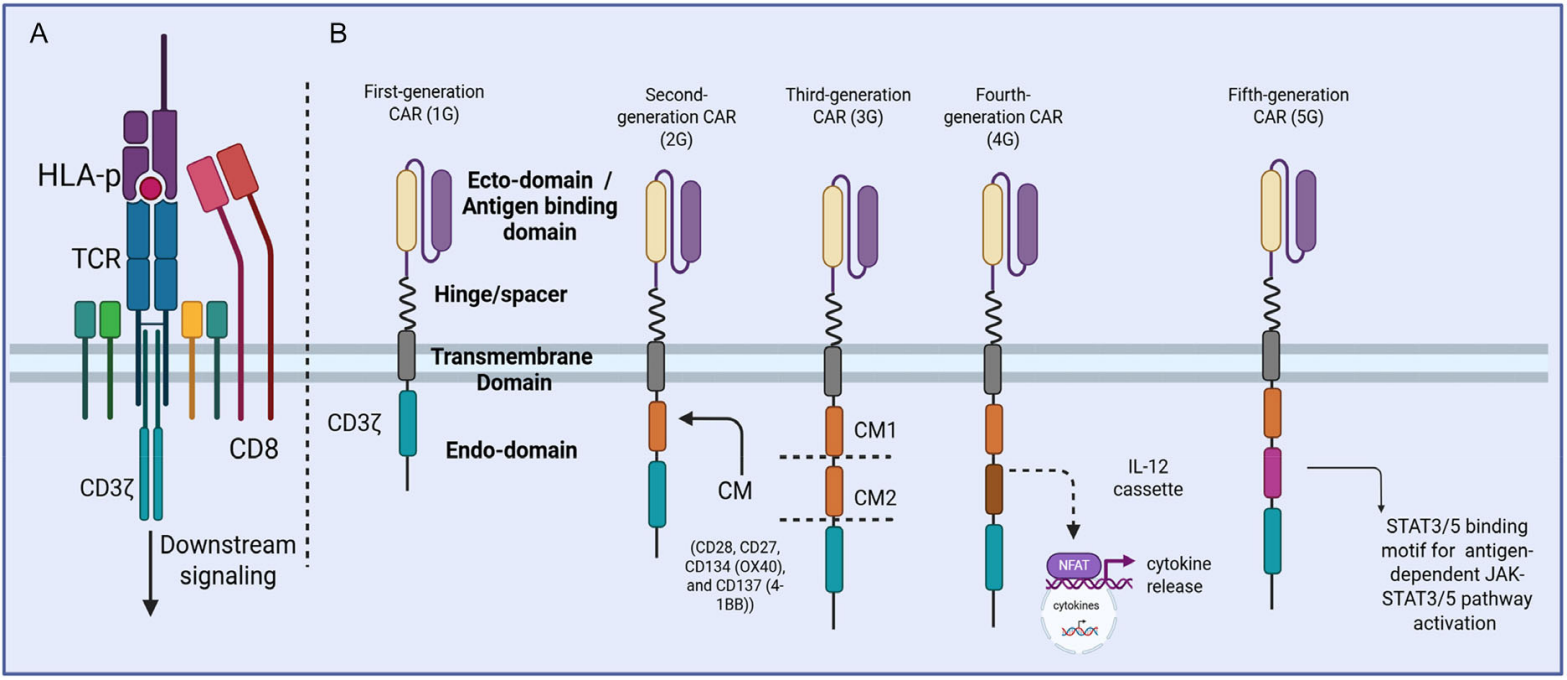
Evolutional development of CAR‐T cell products. A) TCR structure and molecular components. B) Five generations of CAR‐T cell structures. First‐generation CAR‐T cells only contain CD3ζ‐derived signaling modules. Second‐generation CAR‐T cells contain a CD3ζ‐derived signaling module and a costimulatory module (CM). Third‐generation CAR‐T cells contain a CD3ζ‐derived signaling module and two CM, including 4‐1BB, CD28, OX40 or ICOS. Fourth‐generation CAR‐T cells contain a CD3ζ‐derived signaling module, a CM, and a cytokine (such as IL‐12) producing module. Fifth‐generation CAR‐T cells consist of signaling motifs that activate pathways such as JAK‐STAT. TCR, TCR; HLA‐p, human leukocyte antigen and peptide. Created with BioRender.com.

In recent years, CAR‐T cell therapy (CTT) has emerged as one of the most rapidly advancing and widely applied cancer treatments.^[^ ^27^, ^28^ ^]^ In this approach, T cells are engineered with synthetic receptors containing an scFv antigen‐binding domain fused to intracellular activation domains. This design mimics TCR function but enables MHC‐independent recognition of tumor surface antigens and effective tumor cell killing.^[^ ^29^ ^]^ CAR‐T cell therapies have already evolved through five generations. The first generation of CAR‐T cells consists of extracellular antigen recognition domains and intracellular CD3ζ. However, the lack of T cell costimulatory domains cannot stimulate T cell proliferation in vivo, leading to a low antitumor effect. The second‐generation CARs incorporate one costimulatory domain (e.g., CD28 or 4‐1BB), enhancing T cell survival and functions. The third‐generation CARs combine two costimulatory domains for further improved potency. Incorporating additional costimulatory domains into the second‐ and third‐generation CAR‐T cells enhances their persistence in patients and improves therapeutic efficacy; however, the strong and continuous signaling from these costimulatory domains can cause overstimulation, thereby increasing their susceptibility to inhibitory signals and accelerating T cell exhaustion. The fourth‐generation CARs, also known as TRUCKs (T cells redirected for antigen‐unrestricted cytokine‐initiated killing), are engineered to secrete cytokines like interleukin (IL)‐12 to enhance T cell proliferation and survival while modulating the tumor microenvironment (TME). However, they often face challenges including systemic cytokine toxicity, limited control over cytokine expression, and reduced T cell persistence due to exhaustion. The fifth‐generation CARs integrate signaling motifs that activate pathways such as JAK‐STAT, enabling more physiologically controlled immune responses (**Figure** **1**). Unlike earlier generations, fifth‐generation CAR‐T cells include an additional membrane receptor—typically an IL‐2 receptor domain—that activates JAK/STAT signaling in an antigen‐dependent manner and enhances CAR T cell proliferation and persistence without systemic cytokine release. Moreover, the fifth‐generation designs explore additional safety features such as drug‐inducible OFF‐switches for CAR depletion and ON‐switches for controlled activation, offering a broader therapeutic window and improved safety profile.^[^ ^30^, ^31^, ^32^ ^]^

Clinically, the first CTT approved by the US Food and Drug Administration (FDA) in 2017 was a product called Kymriah, released by Novartis. It targets CD19 and treats B cell malignancies, demonstrating a promising efficacy. Subsequently, several CAR‐T cell products have been approved for the treatment of acute lymphoblastic leukemia, multiple myeloma, and various forms of lymphoma (**Table** **1**). Currently, all FDA‐approved CAR‐T therapies use the second‐generation CAR constructs with either CD28 or 4‐1BB costimulatory domains. While demonstrating its efficacy in eradication of hematological malignancies,CTT, however, faces noted challenges in the treatment of solid tumors.^[^ ^22^, ^33^ ^]^ As of September 30, 2025, a total of 286 clinical trials of CTT for solid tumors have been registered worldwide on ClinicalTrials.gov, but none have received FDA approval to date. Early trials using the first‐generation CAR‐T cells targeting neuroblastoma, renal cancer, and ovarian cancer showed limited clinical activity, largely due to insufficient T cell expansion and persistence in vivo. More recent studies on the second‐ and third‐generation CAR‐T cells show some encouraging outcomes.^[^ ^34^ ^]^ However, the majority of CTT trials targeting solid tumors are still in early clinical phases, and their efficacy remains limited, often accompanied by on‐target, off‐tumor toxicities that can lead to severe adverse effects.

**Table 1:** FDA‐approved CAR‐T cell therapies.

CAR‐T name	Target	Costimulatory domain	Disease	Complete response rate	Overall response rate	Grade 3 or higher CRS	Grade 3 or higher ICANS	Approval year	Reference
Tisa‐cel	CD19	4‐1BB	B‐ALL, DLBCL	73% 40%	90% 52%	77% 22%	40% 12%	2017	[177, 178, 179, 180]
Axi‐cel	CD19	CD28	LBCL	54%	82%	13%	28%	2017	[181, 182]
Brexu‐cel	CD19	CD28	MCL, ALL	59% 56%	81% 71%	15% 24%	31% 25%	2020	[183, 184]
Liso‐cel	CD19	4‐1BB	LBCL, CLL	53%	73%	42%	30%	2021	[185]
Ide‐cel	BCMA	4‐1BB	MM	39%	76%	6%	3%	2021	[186]
Cilta‐cel	BCMA	4‐1BB	MM	67%	97%	4%	9%	2022	[187]
Obe‐cel	CD19	4‐1BB	B‐ALL	55%	77%	2%	7%	2024	[188]

Note: BCMA, B cell maturation antigen; A‐ALL, B cell precursor acute lymphoblastic leukemia; DLBCL, diffuse large B cell lymphoma; LBCL, large B cell lymphoma; MCL, mantle cell lymphoma; ALL, acute lymphoblastic leukemia; LBCL, large B cell lymphoma; CLL, chronic lymphocytic leukemia; MM, multiple myeloma.

## 2 CAR‐T Immunotherapy and Challenges in the Treatment of Solid Tumor

**Figure 2:**
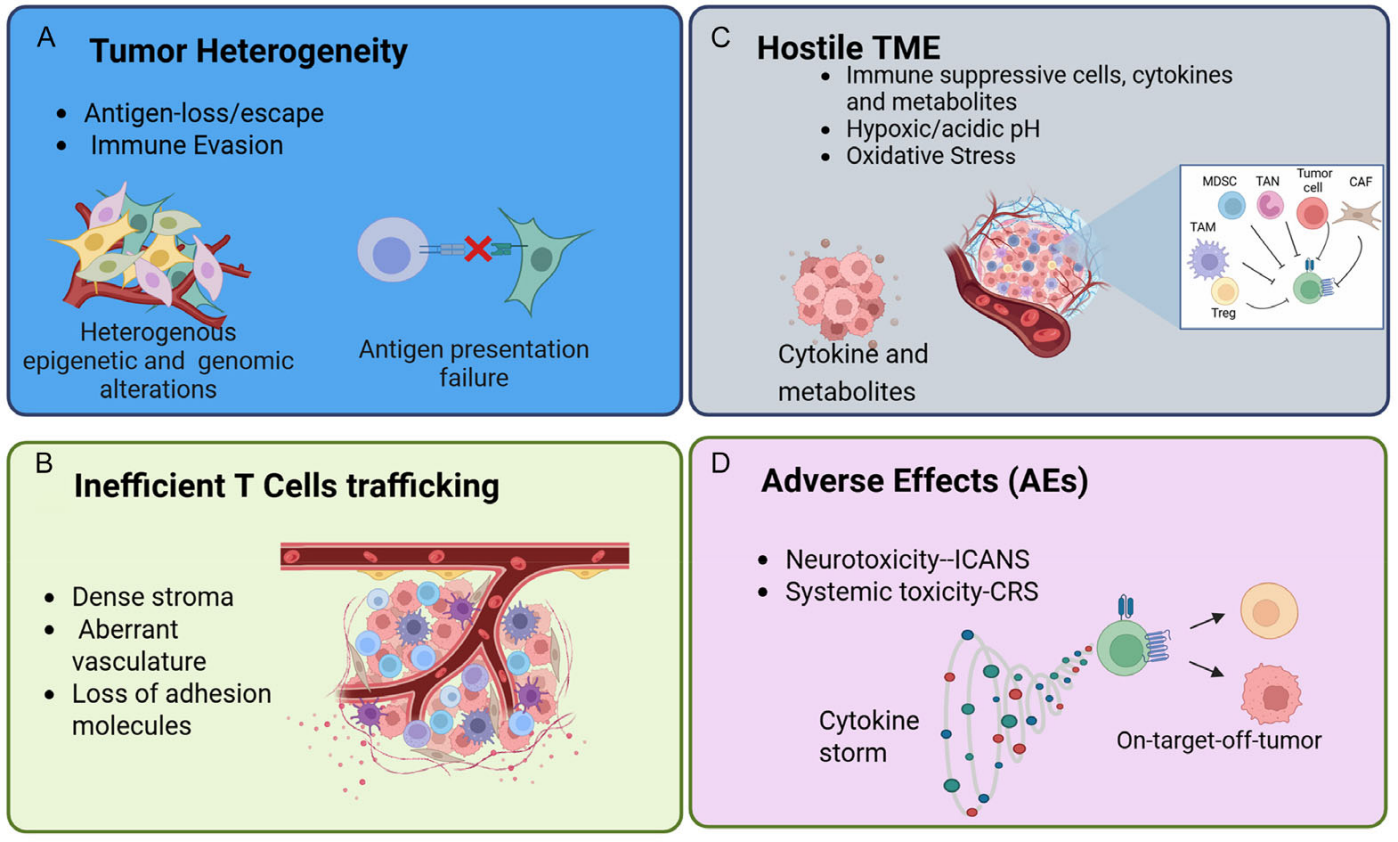
Challenges of CTT in solid tumors. This illustration highlights key barriers that limit the efficacy of CTT in solid tumors. A) Tumor heterogeneity presents a major challenge, as antigen‐loss variants and immune evasion mechanisms—driven by heterogeneous epigenetic and genomic alterations—can result in antigen presentation failure and escape from T cell recognition. B) Inefficient T cell trafficking poses another obstacle, with dense stromal architecture, abnormal vasculature, and the loss of adhesion molecules impairing T cell infiltration into tumor sites. C) The hostile TME further suppresses CAR T cell activity through the presence of immunosuppressive cells, cytokines, and metabolites, as well as physical conditions such as hypoxia, acidic pH, and oxidative stress. D) Adverse effects, including neurotoxicity (ICANS) and systemic toxicity (CRS), as well as potential on‐target off‐tumor activity, can damage healthy tissues. Figure created with BioRender.com.

Notably, CAR‐T therapy has lagged behind in the treatment of solid tumors and continues to face challenges (**Figure** **2**), including A) heterogeneous expression of tumor‐specific antigens (TSA), B) inefficient CAR‐T cell trafficking and infiltration into tumor sites, C) immunosuppressive TME, and D) potential clinical adverse effects. Tumor‐associated antigens (TAAs), which are overexpressed on the surface of cancer cells, and tumor‐specific antigens (TSAs), which are exclusively expressed by cancer cells, play a crucial role in regulating the effectiveness of CAR‐T cell therapies.^[^ ^35^ ^]^ These antigens allow the receptors of engineered CAR‐T cells to specifically target to cancer cells and subsequently eliminate them while sparing the healthy tissues to minimize the off‐target effects. However, tumor antigens in solid tumor typically display a large heterogeneity in phenotype with consequent variation of antigen expression. Intratumoral heterogeneity of antigen expression primarily results from continuous evolution of tumor cells. Such a heterogeneity typically results from antigen mutation, antigen downregulation or loss, and clonal evolution referred to as “antigen escape” from immune responses.^[^ ^36^, ^37^ ^]^ The circumstance with limited infiltration of CAR‐T cells in solid tumors is partially due to the presence of dense, nonmalignant stroma and aberrant vasculature at the tumor site. For CAR‐T cells to effectively target tumor cells, they must exit the tumor vasculature, penetrate the dense extracellular matrix (ECM) of tumor stroma, prior to reaching tumor cells. This process, known as T cell trafficking, involves several key steps, i.e., rolling on the luminal surface of endothelium, adhering to endothelial cells (ECs) through integrins, and ultimately crossing the vasculature wall (extravasation) into the tumor.^[^ ^38^, ^39^ ^]^ In solid tumors, expression of the adhesion molecules like intercellular adhesion molecule (ICAM)‐1 and vascular cell adhesion molecule (VCAM)‐1 on ECs is often suppressed by the proangiogenic factors such as vascular endothelial growth factor (VEGF) and fibroblast growth factor (FGF), a condition known as EC anergy, which significantly hinders immune cells from infiltration.^[^ ^40^ ^]^ In addition, the vasculature networks in solid tumors are irregular, disorganized, and leaky, leading to uneven blood flow and high interstitial fluid pressure, further obstructing the entry of immune cells.^[^ ^41^ ^]^ Upon reaching to the tumor, CAR‐T cells also experience a hostile and immunosuppressive TME that inhibits their activation while accelerating their exhaustion to reduce the persistence. Notably, TME displays unique pathophysiological attributes like hypoxia, acidic pH, cytokines, metabolites, abnormal vasculature, and the presence of various immunosuppressive cells, including regulatory T cells (Tregs), tumor‐associated macrophages (TAMs), and myeloid‐derived suppressor cells (MDSCs), along with stromal cells like ECs and CAFs.^[^ ^42^ ^]^ These collectively impair the antitumor activity of CAR‐T cells. While revolutionizing the cancer treatment, CTT also presents some clinical challenges, particularly the adverse effects caused by CAR T cells like cytokine release syndrome (CRS) and immune effector cell‐associated neurotoxicity syndrome (ICANS).^[^ ^35^, ^43^ ^]^ Cytokines such as IL‐1 and IL‐6 are the key players of the abovementioned toxicities and could be the potential therapeutic targets.^[^ ^44^, ^45^, ^46^ ^]^ Besides, off‐target effects of CAR‐T cells may also cause organ dysfunction, neurological symptoms, and anaphylaxis, which can threaten the patients’ lives. Clearly, development of proper monitoring and management modality after CAR‐T cell infusion becomes highly crucial, especially for those patients experiencing immune‐related toxicities (irAEs) with predisposition to infections.^[^ ^47^ ^]^

The multifaceted challenges limiting the efficacy of CAR‐T and other cell‐based cancer therapies in solid tumors underscore the urgent need for advanced delivery and modulation strategies. In this regard, nanomaterials offer a highly promising platform to enhance the safety, precision, and therapeutic performance of CTTs. Owing to their tunable physicochemical properties and high surface area‐to‐volume ratio, nanoscale systems can be engineered to improve CAR‐T cell expansion, trafficking, and activation within the hostile TME. For instance, nanoparticles functionalized with tumor‐homing ligands or chemokine‐mimetic peptides can facilitate targeted recruitment of CAR‐T cells to tumor sites and improve extravasation through abnormal vasculature. In parallel, nanocarriers capable of locally releasing cytokines, checkpoint inhibitors, or metabolic modulators can remodel the immunosuppressive TME, reverse T cell exhaustion, and enhance persistence without systemic toxicity. Additionally, nanomaterial‐based scaffolds or artificial antigen‐presenting platforms can serve as ex vivo tools to potentiate CAR‐T cell proliferation and memory differentiation prior to reinfusion. Furthermore, smart nanomaterials responsive to pH, enzymatic activity, or redox gradients within the TME enable on‐demand and localized immunomodulation, reducing the risk of CRS and off‐target immune activation. Collectively, these emerging nanotechnological strategies hold immense potential to address the major bottlenecks in solid tumor CTT—improving tumor targeting, infiltration, and functional persistence—thereby paving the way toward safer, more effective cellular immunotherapies.

## 3 Promise of Nanomedicine in Overcoming the CTT of Solid Tumor

In recognition of the challenges associated with CTT for solid tumors, utilizing nanomaterials to boost various aspects of CAR‐T‐associated challenges has received increasing attention for their noted capabilities. Nanomaterials used to enhance CTT can exert their effects through multiple mechanisms, offering a multifaceted strategy to improve therapeutic outcomes. The following sections discuss in detail the representative use of selected nanomaterials in addressing some of the key challenges associated with the development of safe and effective CAR T cell therapies for solid tumors.

### 3.1 Addressing Antigen Heterogeneity and Escape in Solid Tumors

**Figure 3:**
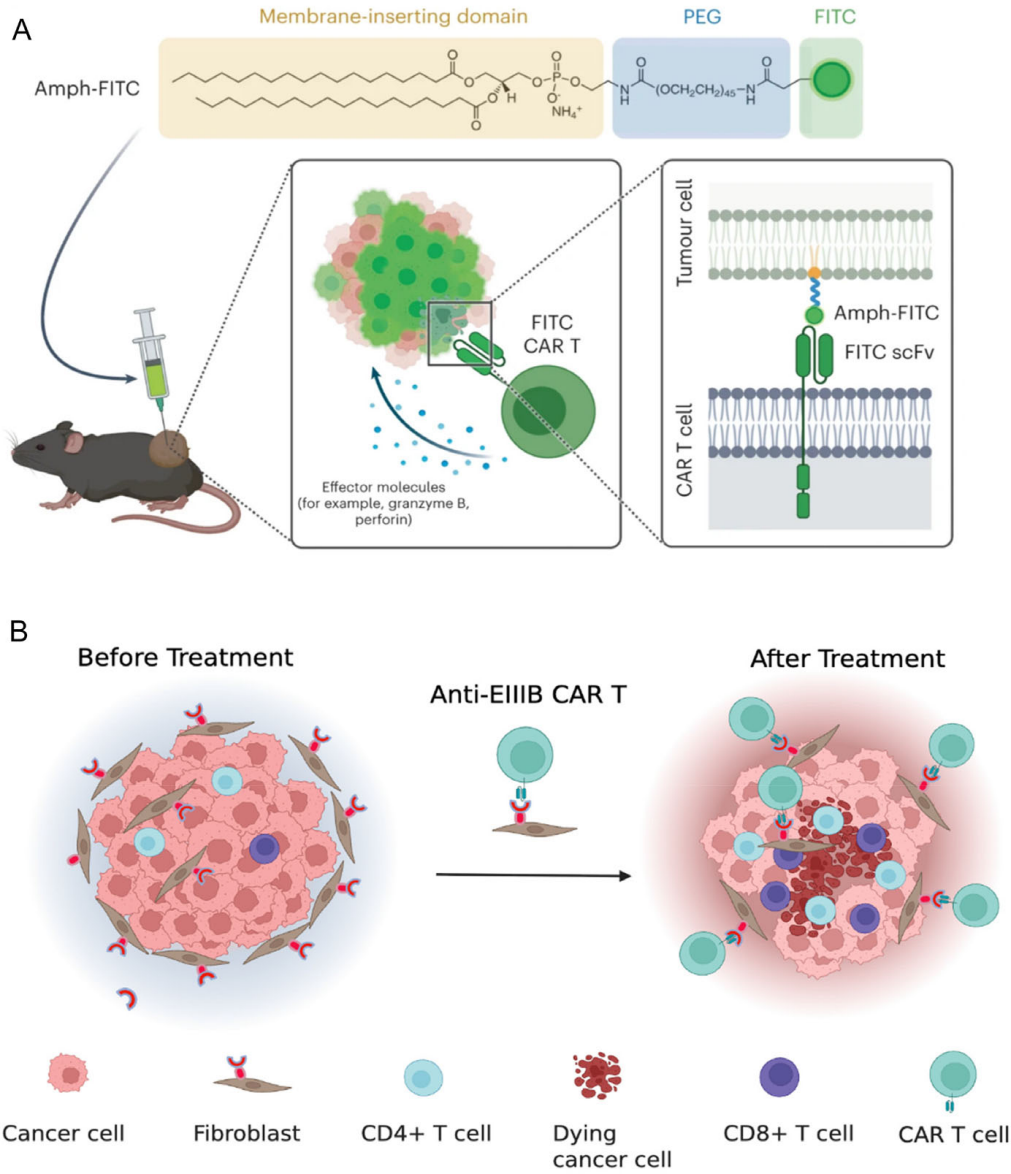
Nanotechnology‐based strategies to overcome antigen heterogeneity in solid tumors for CTT. A) Tumor cells are labeled with universal CAR T cell‐recognizable tags, such as FITC, to enhance recognition by engineered CAR T cells. Adapted under the terms of the CC‐BY Creative Commons Attribution 4.0 International license (https://creativecommons.org/licenses/by/4.0).^[^ ^48^ ^]^ Copyright 2023, the Authors, Published by Springer Nature. B) Development of CAR T cells targeting abnormally expressed antigens in stromal components, such as EIIIB found in fibroblasts, to broaden therapeutic targeting. Created with BioRender.com.

During CTT, TSA targets are specially required for CAR T cells to interact with to achieve efficient tumor‐killing; however, the loss or aberration of such TSAs often leads to CTT resistance or relapse. To overcome the lack of TSA for CAR T cells, an applauding strategy is to modify the tumor cells with universal CAR T cell‐recognizing tags, which can be achieved by inserting the corresponding ligand molecules into tumor cell membrane or therapeutically inducing the expression of exogenous antigens on tumor cells (see **Figure** **3**). For example, Zhang et al. designed a “universal” yet CAR T cell‐recognizing nanotag and then inserted it into solid tumor cell membranes for the CAR.^[^ ^48^ ^]^ Amphiphilic poly(ethylene glycol) (PEG)‐lipid (nanomaterial conjugates) conjugated to fluorescein isothiocyanate (FITC) (“amph‐ligand”) was used to decorate the tumor cell membrane, which could be recognized by CAR T cells bearing FITC‐specific chimeric receptors and trigger CAR T cell activation and proliferation to kill the FITC‐decorated tumor cells, leading to tumor regression in mouse tumor xenograft models.^[^ ^48^ ^]^ Such “amph‐ligand” conjugates exhibit several distinct advantages, including 1) manipulating the pharmacokinetics and biodistribution of linked molecules, 2) spatially controlling the display of conjugated molecules (i.e., FITC) on the cell surface upon insertion of the lipid tails of the conjugates into the cell plasma membranes without toxicity in vitro and in vivo, and 3) efficiently trafficking into draining lymph nodes (LNs) and decorating the surfaces of antigen‐presenting cells (APCs) following parenteral injection. Clearly, such a strategy is highly appealing for the possible selection of antigens particular to cancer cells without the concern of cross targeting healthy tissue. Clearly, induction of antigen on the transduced tumors for CAR T cell attack promotes endogenous T cell responses that ameliorate antigen‐loss escape and provide systemic antitumor immunity.

Another compelling strategy to bypass the need for TSAs is to design CAR T cells targeting the TME and subsequently suppress the tumor progression. For instance, Xie et al. developed the variable single domain of heavy‐chain only antibodies (known as VHH or nanobody) for the EIIIB splice variant of fibronectin (NJB2) to specifically target the tumor stroma and vasculature by recognizing the EIIIB^+^ fibronectin splice variant, typically presenting in various tumor types and neo‐angiogenic cells.^[^ ^49^ ^]^ Different from single‐chain variable fragment (scFvs, both heavy and light chains), heavy‐chain‐only VHH antibodies are small, highly stable, very accessible, and low immunogenic yet with high affinities comparable to scFvs. Building on these advantages, CAR‐T cells engineered with EIIIB^+^‐recognizing VHH domains were developed and shown to effectively reduce tumor growth in the B16 melanoma model. Attacking the tumor stroma and/or the neovasculature may not only help to establish a local inflammatory response that benefits subsequent immune recognition in a vaccinal fashion, but it may also enhance access to the tumor for otherwise impermeant drugs in difficult‐to‐treat cancers.

### 3.2 Enhancing CAR T Cell Trafficking and Tumor Infiltration

As widely recognized, TME is a highly dynamic and complex niche composed of tumor vasculature, connective tissue, infiltrating immune cells, and a dense ECM. Notably, the ECM within the TME is rich in structural proteins such as collagen and is further modulated by an array of bioactive molecules (e.g., growth factors, cytokines, and MMPs). The intricate ECM network not only provides mechanical support but also orchestrates the key biochemical cues to regulate tumor progression. Crucially, the stiffness of the ECM can be significantly elevated due to reciprocal interactions between cancer cells and stromal cells. Such a pathological ECM stiffening elevates intratumoral interstitial fluid pressure (IFP), disrupts transcapillary exchange, and impedes the effective penetration and distribution of therapeutic agents. In addition, the altered mechanical and biochemical properties of the ECM create a formidable physical barrier to immune cell infiltration and activation, contributing to immune evasion and resistance to immunotherapy. Using nanomaterials to promote ECM degradation or to enable direct delivery of CAR‐T cells into the TEM has emerged as a promising strategy to overcome the physical barriers imposed by the ECM, thereby enhancing therapeutic efficacy.

#### 3.2.1 Enhanced Cell Delivery and Functions

##### Polymerized Alginate Macroporous Scaffold

**Figure 4:**
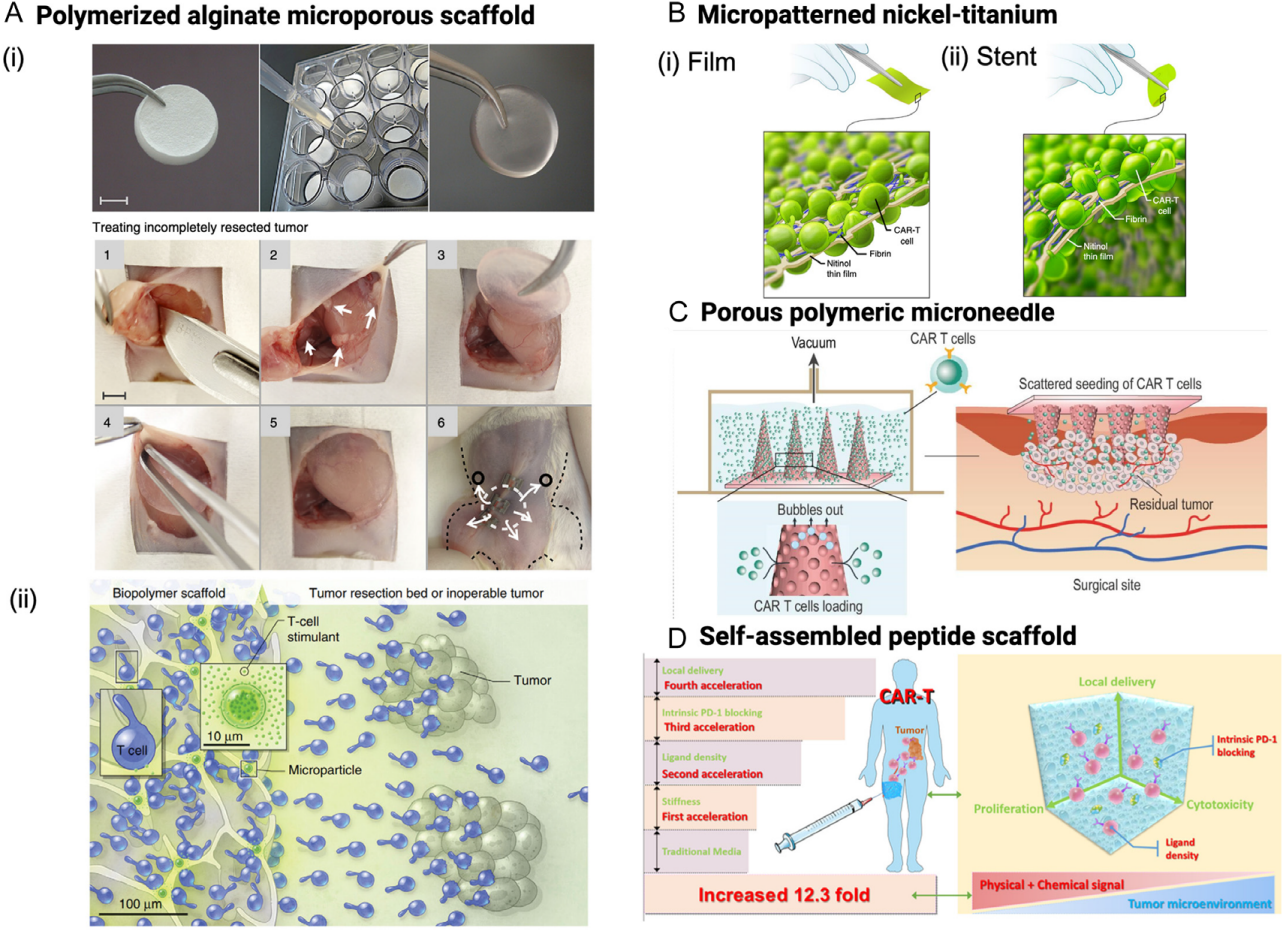
Nanoscaffold‐based platforms for enhancing localized delivery and efficacy of CAR T cells in solid tumors. A) Polymerized alginate macroporous scaffold—designed to support CAR T cell viability and expansion, enabling sustained release directly into the TME. Adapted with permission.^[^ ^175^ ^]^ Copyright 2015, Springer Nature. B) Micropatterned nickel‐titanium (nitinol) films and self‐expandable stents—engineered to deliver CAR T cells into tumors while physically maintaining access to the tumor core and resisting tissue ingrowth. Adapted with permission.^[^ ^51^ ^]^ Copyright 2020, Springer Nature. C) PMN patch—allows minimally invasive, localized CAR T cell delivery through the skin, facilitating direct infiltration into tumor tissue. Adapted under the terms of the CC‐BY Creative Commons Attribution 4.0 International license (https://creativecommons.org/licenses/by/4.0).^[^ ^176^ ^]^ Copyright 2021, the Authors, Published by Oxford University Press on behalf of China Science Publishing & Media Ltd. D) Self‐assembled peptide hydrogel—mimics the ECM to enhance CAR T cell retention, proliferation, and antitumor activity within the immunosuppressive TME. Adapted with permission.^[^ ^56^ ^]^ Copyright 2022, American Chemical Society.

To address the challenges of lymphocyte trafficking to tumors and limited T cell expansion within the immunosuppressive TME, the *Stephan* group engineered a macroporous alginate‐based scaffold designed to deliver T cells and enhance their function in the treatment of incompletely resected or inoperable tumors (**Figure** **4**A). With the presence of interconnected macropores, the alginate‐based scaffold functionalized as an active reservoir supporting T cell proliferation and enabling their sustained release as the scaffold gradually degraded (Figure 4A(ii)).^[^ ^50^ ^]^ To enhance T cell release from the scaffolds, a collagen‐mimetic peptide (GFOGER) was conjugated to alginate using carbimide chemistry, creating a biomimetic scaffold that leveraged lymphocytes’ inherent ability to migrate along collagen fibers via α2β1 integrin. Architecturally, the scaffold incorporated interconnected voids that housed vesicles containing IL‐15/IL‐15Rα fusion proteins and porous silica cores. These vesicles were surface‐functionalized with stimulatory antibodies (αCD3, αCD28, and αCD137) to induce robust TCR clustering and activation. This design enabled localized and sustained T cell stimulation at the tumor resection site, where T cell activation is typically inefficient, leading to a striking 167‐fold increase in T cell accumulation compared to direct infusion of pre‐stimulated cells. Unlike systemically delivered T cells, which often lose effector phenotypes en route to and within the tumor, scaffold‐activated T cells maintained a potent effector profile at the tumor site. In a murine model of peritoneal serous ovarian cancer, scaffold‐enabled delivery led to a 12‐fold increase in T cell accumulation in the peritoneal cavity, highlighting the promise of such a platform for enhancing CTT efficacy in solid tumors. This work underscores the therapeutic potential of in situ T cell activation through biomaterial‐based delivery systems.

##### Micropatterned Nickel‐Titanium Film and Stent

Micropatterned nickel‐titanium (nitinol) meshes, valued for their biocompatibility, superelasticity, and shape‐memory behavior, have emerged as innovative platforms for CAR T cell delivery (Figure 4B).^[^ ^51^ ^]^ These dynamic materials can be engineered into deployable stents or thin films (thickness of 10 μm) that not only conform to complex anatomical sites but also mechanically disrupt the dense ECM, thereby enhancing immune cell infiltration into otherwise inaccessible tumor tissues. To better support the adhesion and activation of T cells, the micropattern substrates were functionalized with adhesion (fibrin coating) and stimulatory antibodies (anti‐CD3, anti‐CD 28 and anti‐CD137) that are known to bind to and activate T cells for rapid expansion. As demonstrated in a murine model of nonresectable ovarian cancer, nitinol thin films loaded with T cells facilitated rapid expansion and high‐density, localized delivery of CAR T cells to the tumor bed, resulting in significantly improved survival outcomes. Moreover, self‐expanding stents coated with T cell‐laden films, when implanted into subcutaneous tumors, delayed tumor ingrowth and prolonged stent patency, further underscoring the therapeutic utility of this approach. Despite these promising outcomes, key engineering challenges remain in achieving microscale geometrical precision, maximizing cell loading while minimizing the biomaterial footprint, and maintaining adequate gas and nutrient exchange to support cell viability. While the need for scaffold retrieval post‐treatment may pose clinical constraints, the existing clinical use of nitinol in FDA‐approved devices could streamline its translational pathway, making it a compelling candidate for integration into next‐generation CAR T cell therapies.^[^ ^52^ ^]^

##### Porous Microneedle (PMN) Patch

PMN patches offer a minimally invasive strategy for overcoming the formidable barriers posed by dense tumor architectures, while simultaneously enhancing patient compliance and eliminating the pain typically associated with conventional injections. In a notable example, Li et al. engineered a polymeric PMN patch capable of locally dispersing CAR T cells at the tumor site, significantly improving cellular seeding, infiltration, and therapeutic engagement (Figure 4C).^[^ ^53^ ^]^ In a melanoma model, PMN‐mediated delivery led to a three‐fold increase in CAR T cell proliferation and markedly enhanced in situ persistence compared to conventional systemic administration. These findings underscore the potential of the PMN platform to augment CAR CTT by enabling localized, sustained, and efficient immune cell delivery. Complementary studies further support microneedles (MNs) as a versatile platform to bypass intradermal injections, facilitating controlled and targeted delivery of vaccines, cells, or biologics with minimal invasiveness and high translational promise.^[^ ^54^, ^55^ ^]^

##### Self‐Assembled Peptide Hydrogel

Beyond the need to penetrate a heterogeneous and immunosuppressive ECM, achieving robust CAR T cell expansion in vivo, without prolonged ex vivo culture, is critical, as extended manipulation can compromise the function and phenotype of effector T cells. To address this limitation, Jie et al.^[^ ^56^ ^]^ developed a self‐assembled peptide hydrogel engineered to simultaneously modulate mechano‐transduction and deliver supportive chemical cues (Figure 4D). This biomimetic matrix served not only as a structural support but also as an immunomodulatory niche that fostered CAR T cell proliferation and function within TME. In parallel, the team engineered CAR T cells to secrete a PD‐1‐blocking scFv, enabling autocrine and paracrine checkpoint inhibition. This dual approach significantly enhanced CAR T cell expansion, persistence, and cytotoxicity by overcoming both intrinsic exhaustion and extrinsic immunosuppressive cues within the tumor immune microenvironment (TIME). Collectively, this strategy represents a powerful platform for improving CTT by enabling localized, sustained activation and remodeling the TIME to better support durable antitumor immunity.

#### 3.2.2 Hard‐to‐Infiltrate ECM

Degradation of the tumor ECM has long been recognized as a pivotal strategy to enhance immune cell infiltration and therapeutic penetration in solid tumors. The ECM in most solid tumors is characterized by excessive deposition of HA and collagen, which together create a dense, fibrotic network that elevates interstitial fluid pressure, restricts vascular perfusion, and physically obstructs immune cell migration. As recognized, HA forms a highly hydrated, gel‐like matrix that traps macromolecules and impedes diffusion, whereas collagen, primarily type I and III fibers, contributes to the mechanical stiffness and structural rigidity of the tumor stroma. Enzymatic degradation of these barriers, particularly through hyaluronidase (HAase) and collagenase, has been widely used to remodel the TME and improve tissue accessibility to therapeutic agents and immune effector cells. However, the short half‐life, systemic instability, and off‐target toxicity of free enzymes have limited their clinical translation. Nanotechnology‐based delivery platforms have emerged as powerful tools to overcome these constraints by improving enzyme stability, bioavailability, and tumor‐specific accumulation.

Recent innovations, such as HAase‐ or collagenase‐loaded nanogels (NG), liposomes, or polymeric nanoparticles (PNPs), as well as enzyme nanoconjugates integrated onto CAR‐T cell membranes, have demonstrated remarkable potential to achieve localized and sustained ECM degradation, thereby promoting deeper immune cell infiltration into solid tumors.^[^ ^57^, ^58^, ^59^, ^60^, ^61^, ^62^ ^]^ These nanoplatforms are often engineered to respond to tumor‐specific cues such as acidic pH, elevated glutathione (GSH), or matrix metalloproteinase (MMP) activity, allowing for on‐demand enzyme release precisely within TME^[^ ^63^ ^]^ For example, HAase‐loaded NGs conjugated to CAR‐T cell membranes enable the enzymatic degradation of pericellular HA in situ, creating transient “pathways” that facilitate T cell migration through the dense stromal matrix without compromising cell viability.^[^ ^64^ ^]^ Similarly, collagenase‐loaded nanoparticles have been designed to soften fibrotic tumor stroma, reduce interstitial pressure, and improve vascular perfusion, thereby synergizing with CAR‐T‐mediated cytotoxicity.^[^ ^65^, ^66^ ^]^ Notably, bioorthogonal and NG‐based HAase‐armed CAR‐T systems, as demonstrated by Zhao et al.,^[^ ^67^ ^]^ represent a landmark advancement in this domain. In their study, a bioorthogonal click chemistry strategy was used to conjugate HAase‐containing NGs onto CAR‐T cell surfaces, ensuring stable attachment and sustained enzymatic activity during circulation and tumor infiltration. This approach effectively degraded intratumoral HA, enhanced CAR‐T motility, and significantly improved tumor regression and survival in murine solid tumor models without systemic toxicity. While single‐enzyme approaches have shown promise, the codelivery of both HAase and collagenase as a single nanoplatform represents a prospective, synergistic approach to degrade the physical tumor barrier.^[^ ^68^, ^69^, ^70^ ^]^ However, no studies exist for codelivery of the abovementioned enzymes to enhance CTT. The design of such nanoplatforms will require prudent optimization to ensure robust enzyme encapsulation (or via click conjugation) and controlled release kinetics with minimal impact on CTT function and cell viability. For example, size‐changeable collagenase‐modified nanoscavenger suggests strategies for enhancing CTT. Concepts from this study, such as targeted enzyme release and mechanisms for increased retention, can inform future designs of modified CAR T cells or combined therapies to ameliorate tumor infiltration.^[^ ^69^, ^71^ ^]^ Moreover, although not nanomedicine, the demonstrated benefit of tumor‐restricted ECM debulking can inform the engineering of pH/MMP‐gated enzyme nanocarriers and backpack approaches on CAR‐T to improve trafficking in fibrotic tumors.^[^ ^72^ ^]^ While numerous nanoplatforms have investigated the utilization of ECM debulking enzymes, at this stage, the two fields have yet to fully merge.^[^ ^73^, ^74^ ^]^

To date, only a subset of ECM‐degrading enzymes has been directly integrated into CTT or T cell‐based therapies. These include HPSE (heparinase),^[^ ^75^ ^]^ MMP‐7/8,^[^ ^76^, ^77^ ^]^ neuraminidase (CpNA),^[^ ^78^ ^]^ kynureninase (KYNU),^[^ ^79^ ^]^ and β‐lactamase (in SEAKER systems),^[^ ^80^ ^]^ each demonstrating improved tumor infiltration or antitumor potency in preclinical models. FAP (fibroblast activation protein), a stromal protease, represents a distinct but complementary strategy in which CAR T cells target the enzyme rather than utilize it, illustrating an antistromal variant of ECM modulation. Targeting CAFs via FAP has shown mixed preclinical efficacy and scFv‐dependent toxicity (cachexia, bone effects), while intrapleural anti‐FAP CAR‐T in a small cohort was well tolerated but requires broader efficacy evaluation (NCT01722149; ongoing NCT03932565). Neutralizing CAF‐derived CXCL12 (e.g., CXCR4 inhibition ± anti‐PD‐L1) rapidly increases intratumoral T cell accumulation in PDAC models.^[^ ^81^ ^]^ Other ECM‐active enzymes—such as stromelysins, plasmin, elastase, and cathepsins—remain underexplored in CAR T engineering due to concerns about systemic toxicity or insufficient control of activity.^[^ ^57^, ^58^, ^82^, ^83^ ^]^ Studies show selective incorporation or conjugation of ECM‐modulating enzymes can reprogram the tumor‐associated ECM, laying the foundation for arming next‐generation nanosymbiont CAR T systems.^[^ ^59^, ^60^, ^74^ ^]^

Collectively, these enzyme–nanomaterial hybrid systems exemplify a new generation of ECM‐responsive immunomodulatory nanotechnologies that actively reshape the tumor milieu to enhance CAR‐T cell infiltration, persistence, and therapeutic efficacy in solid tumors.^[^ ^61^, ^62^, ^84^ ^]^ Together, these approaches highlight the promise of enzyme–nanomaterial synergy in modulating the ECM to overcome one of the most formidable barriers to effective CAR‐T therapy in solid tumors.

### 3.3 Modulating the TME to Enhance CAR T Cell Function

**Figure 5:**
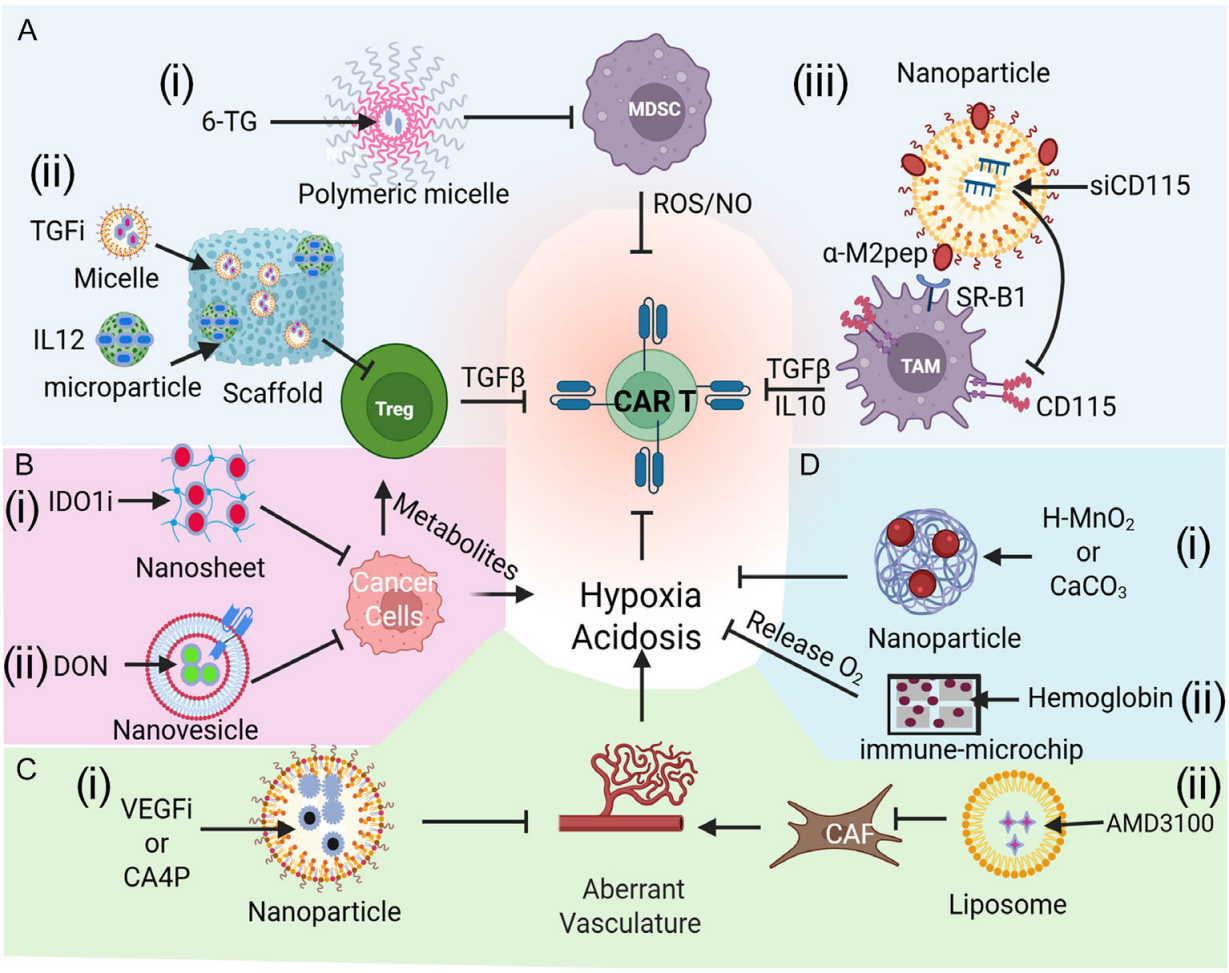
Nanomedicine‐based strategies for targeting immunosuppressive factors in the TME to enhance CAR T cell activity. A. Targeting suppressive immune cells. i) Polymeric micelles are used to deliver 6‐thioguanine (6‐TG) for the selective depletion of MDSCs. ii) A biodegradable macroporous scaffold implanted peritumorally releases a TGF‐β inhibitor to suppress regulatory T cells (Tregs), along with codelivered IL‐2 to promote CAR T cell proliferation. iii) A dual‐targeting nanocarrier, incorporating both an SRB1‐targeting α‐peptide and M2 macrophage‐specific M2Pep, delivers siRNA selectively to M2‐like TAMs. B. Targeting tumor metabolites: Inhibition of immunosuppressive tumor metabolites via nanosheet‐based delivery of an indoleamine 2,3‐dioxygenase 1 (IDO1) inhibitor (i) and NV‐based delivery of the glutamine antagonist 6‐diazo‐5‐oxo‐L‐norleucine (DON) (ii). C. Targeting aberrant vasculature. i) Nanoparticle‐mediated delivery of VEGF inhibitors or the EC inhibitor CA4P to normalize abnormal tumor vasculature. ii) Liposome‐mediated delivery of AMD300 to target CAFs, contributing to vascular normalization. D. Targeting the hypoxia and acidic TME. Reversal of hypoxia and acidic TME conditions using pH‐responsive manganese oxide (H–MnO_2_) nanoplatforms (i) and bicarbonate‐ or calcium carbonate (CaCO_3_)‐based nanocarriers (ii) to neutralize acidic pH. Created with BioRender.com.

As mentioned above, TME is highly heterogeneous and immunologically hostile, posing significant challenges to the efficacy of CAR CTT. Beyond the physical and biochemical barriers of the ECM, the TME is enriched with immunosuppressive cellular populations (e.g., Tregs, TAMs, and MDSCs), which actively suppress CAR T cell function by promoting exhaustion, functional anergy, and apoptosis. Efforts to reprogram this aberrant immune landscape have shown that selective depletion or functional inhibition of these immunosuppressive cell types can reinvigorate both endogenous antitumor immunity and CAR T cell responses. Moreover, these cellular components also amplify immunosuppression through the secretion of noncellular factors such as immunosuppressive cytokines, chemokines, and metabolic enzymes, which further reinforce a tolerogenic, tumor‐permissive microenvironment. Targeting these soluble mediators represents a complementary strategy to modulate the TME, restore CAR T cell functionality, and prevent the onset of T cell exhaustion and dysfunction. Some of the representative utility of nanomaterials to address the immunosuppressive TME is highlighted in **Figure** **5** with more detailed elaboration as below.

#### 3.3.1 Immunosuppressive Cells

MDSCs accumulate in the TME and suppress CAR T cell function by producing immunosuppressive molecules such as arginase‐1, nitric oxide, and reactive oxygen species (ROS), while simultaneously enhancing the recruitment of Tregs and promoting anti‐inflammatory cytokines.^[^ ^85^ ^]^ Thus, targeting MDSCs has emerged as a compelling strategy to restore immune function and enhance CAR T cell activity. Jeanbart et al. addressed this challenge by using polymeric micelles loaded with 6‐thioguanine (6‐TG) to selectively deplete MDSCs in a murine melanoma model (Figure 5A(i)).^[^ ^86^ ^]^ These micelles, composed of polyethyleneimine (PEI) and hyaluronic acid (HA), significantly reduced MDSC populations and enhanced CD8^+^ T cell responses when combined with T cell‐based immunotherapies, demonstrating a promising approach to modulate the TME.

Tregs, a subset of CD4^+^ T cells, are essential for maintaining immune homeostasis but play a detrimental role in cancer by dampening antitumor immunity. Within the TME, Tregs suppress CAR T cell function via the secretion of immunosuppressive cytokines such as IL‐10 and TGF‐β, as well as through direct inhibition of effector T cell activity.^[^ ^87^ ^]^ Their enrichment in solid tumors is closely correlated with poor therapeutic responses. To counteract this, Majedi et al. engineered a biodegradable macroporous scaffold for peritumoral implantation that delivered a combination of immunomodulatory agents: a small‐molecule TGF‐β inhibitor to suppress Treg activity, chemokines, and stimulatory antibodies to recruit and activate effector T cells (Figure 5A(ii)).^[^ ^88^ ^]^ In two aggressive mouse tumor models, this multifunctional implant effectively reduced Treg accumulation, promoted effector T cell infiltration and activation, and induced an “immunological abscopal effect” on distant metastases and established durable immune memory that prevented tumor recurrence, highlighting the potential of this scaffold to synergize with CAR T cell therapy in solid tumors.

TAMs, often skewed toward an immunosuppressive M2‐like phenotype, constitute a dominant immune population in many solid tumors and are major barriers to effective CAR T cell function. These cells secrete inhibitory cytokines (e.g., IL‐10 and TGF‐β), express immune checkpoint ligands (e.g., PD‐L1), and contribute to both physical and metabolic barriers that restrict CAR T cell infiltration and survival.^[^ ^89^ ^]^ Strategies that reprogram TAMs toward a proinflammatory M1‐like phenotype offer a promising route to remodel the TME in favor of antitumor immunity. Qian et al. developed a dual‐targeting nanocarrier incorporating an SRB1‐targeting α‐peptide and the M2‐specific M2Pep to deliver siRNA selectively to M2‐like TAMs (Figure 5A(iii)).^[^ ^90^ ^]^ This targeted approach effectively eliminated immunosuppressive TAMs, reduced IL‐10 and TGF‐β levels, enhanced CD8^+^ T cell infiltration, and improved survival in preclinical models. However, while the role of TAM modulation in general immunotherapy is well documented, its specific impact on CAR T cell dynamics remains an underexplored but promising area of investigation.

Together, these studies underscore the critical need to integrate immunosuppressive cell‐targeting strategies into CAR CTT platforms. Reprogramming or eliminating MDSCs, Tregs, and TAMs can synergistically enhance CAR T cell persistence, trafficking, and cytotoxic function, potentially overcoming one of the most formidable barriers to effective immunotherapy in solid tumors.

#### 3.3.2 Metabolism and Metabolites

Cancer cells reprogram their metabolism to meet increased demands for energy, biosynthesis, and redox balance primarily through aerobic glycolysis, also known as the Warburg effect.^[^ ^91^, ^92^, ^93^ ^]^ This metabolic shift favors glucose uptake and conversion of pyruvate into lactate via lactate dehydrogenase (LDH), even in the presence of oxygen.^[^ ^94^ ^]^ In parallel, tumors exploit amino acid metabolism, consuming large quantities of glutamine and L‐arginine, which are also essential for T cell activation, proliferation, and effector differentiation.^[^ ^91^, ^92^, ^93^ ^]^ While glutamine supports T cell bioenergetics and signaling, L‐arginine promotes survival and cytotoxic function. Conversely, tumors upregulate enzymes such as indoleamine 2,3‐dioxygenase (IDO1), which catabolize tryptophan into kynurenine, a metabolite that suppresses T cell activity and promotes Treg expansion.^[^ ^91^, ^92^, ^93^ ^]^ In this highly competitive and nutrient‐deprived TME, CAR T cells are often metabolically restricted, leading to hypofunction, anergy, and exhaustion, which further compromises the therapeutic efficacy.^[^ ^91^, ^92^, ^93^, ^95^ ^]^

To overcome metabolic constraints, Shao et al. developed the first HA‐functionalized graphene oxide (GO) nanoplatform (HA‐GO) loaded with the IDO1 inhibitor epacadostat to enhance the therapeutic potential of CTT (Figure 5B(i**)**).^[^ ^96^ ^]^ The high surface‐to‐volume ratio and biocompatibility of nanoformulated GO, further improved by HA functionalization, enabled targeted delivery to CD44‐expressing tumor cells as CD44 being a receptor commonly overexpressed in various tumors. HA also served as a capping agent, stabilizing the nanoplatform's size and morphology and prolonging its circulation time in vivo. In a murine model of esophageal squamous cell carcinoma (ESCC), the HA‐GO nanosheets facilitated controlled release of epacadostat, effectively inhibiting kynurenine production and thereby reversing IDO1‐mediated T cell suppression. This metabolic reprogramming enhanced CAR T cell cytokine secretion, effector T cell function, and antitumor activity, demonstrating the promise of nanomaterial‐based strategies to target immunosuppressive metabolic pathways in solid tumors.^[^ ^96^ ^]^

Beyond tryptophan metabolism, the distinct metabolic preferences of immune and tumor cells present additional therapeutic opportunities. While immune cells, including CAR T cells, primarily depend on glucose, tumor cells also heavily rely on glutamine, creating a competitive metabolic bottleneck within TME. Li et al. addressed this by engineering genetically programmable nanovesicles (NVs) expressing high‐affinity anti‐PD‐L1 scFv and encapsulating the glutamine antagonist 6‐diazo‐5‐oxo‐L‐norleucine (DON) (Figure 5B(ii**)**).^[^ ^97^ ^]^ These targeted vesicles (D@aPD‐L1 NVs) selectively delivered DON to PD‐L1^+^ tumor cells, suppressing glutamine metabolism, reversing immunosuppressive signaling, and preventing premature CAR T cell exhaustion. Treatment with these NVs reduced the presence of suppressive immune cell populations, enhanced proinflammatory cytokine production, and promoted robust CAR T cell infiltration and effector activity, ultimately improving the long‐term immune memory. Complementary nanomedicine strategies have also emerged, including gold nanoparticles (AuNPs) functionalized with essential nutrients such as glucose and glutamine, as well as amphiphilic self‐assembling polymeric micelles designed to codeliver metabolic inhibitors like 2‐deoxyglucose (2‐DG).^[^ ^98^, ^99^, ^100^ ^]^ Nutrient‐loaded AuNPs have been investigated as metabolic support vehicles to directly supply CAR T cells with critical substrates in nutrient‐deprived TMEs, thereby enhancing their metabolic fitness and sustaining cytotoxic function during CTT.^[^ ^98^ ^]^ Meanwhile, micellar carriers codelivering 2‐DG and glutamine transport inhibitors aim to disrupt tumor metabolism through dual blockade of glycolysis and glutaminolysis, offering synergistic antitumor effects.^[^ ^99^ ^]^ Together, these metabolic reprogramming platforms represent a promising frontier in cancer immunotherapy. By alleviating nutrient competition, mitigating immunometabolic suppression, and preserving T cell fitness, nanotechnology‐based strategies offer a powerful means to enhance the efficacy, persistence and long‐term success of CAR T cell therapies in solid tumors.

#### 3.3.3 Aberrant Vasculature

Aberrant tumor angiogenesis leads to structurally and functionally abnormal vasculature, contributing to elevated IFP, disrupted perfusion, and poor immunotherapeutic delivery. This disordered blood flow induces a hypoxic, acidic, and nutrient‐deprived TME, impairing cytokine signaling and metabolic fitness of effector T cells and ultimately promoting resistance to immunotherapies.^[^ ^101^ ^]^ To address these challenges, integrating vascular normalization therapies (VNT) with smart nanodrug delivery systems (NDDS) offers a promising multi‐targeted approach. By correcting the leaky, chaotic vasculature typical of tumors, VNT improves oxygenation and reduces hypoxia, creating a more permissive environment for T cell infiltration and function. Numerous studies have demonstrated that antiangiogenic therapies not only normalize tumor vasculature but also synergize with checkpoint blockade and CAR T cell therapies to enhance antitumor responses.^[^ ^102^ ^]^ Preclinical models that incorporate VNT and NDDS represent a compelling next step in improving the efficacy and durability of CTT in solid tumors.

In contrast to normalization, vascular disrupting strategies (VDSs) aim to selectively collapse tumor vasculature, inducing widespread tumor necrosis and promoting a proinflammatory TME. When combined with CAR CTT, vascular disrupting agents (VDAs) can enhance immune cell recruitment and activation, effectively priming the tumor for immune‐mediated destruction. For example, combretastatin A4 phosphate (CA4P), a well‐characterized VDA, selectively targets the ECs of aberrant tumor blood vessels. In a study by Natsume et al., combining CA4P with CAR CTT enhanced tumor necrosis, facilitated antigen release and presentation, and significantly improved T cell infiltration and activation (Figure 5C(i)).^[^ ^103^ ^]^ Preclinical models, including murine xenografts and patient‐derived xenografts (PDXs) of colon and ovarian cancer, demonstrated that CA4P effectively “carves” pathways for CAR T cell trafficking, boosting their intratumoral accumulation and cytotoxic activity.

Aberrant tumor vasculature is further regulated by the CXCL12/CXCR4 signaling axis, which contributes to immune exclusion by facilitating chemokine‐driven recruitment of immunosuppressive cells such as MDSCs and by maintaining fibroblast‐rich, dense stromal barriers. Cancer‐associated fibroblasts (CAFs), a major source of CXCL12, can be targeted to improve immune cell infiltration. Feig et al. showed that depleting CAFs or using the CXCR4 inhibitor AMD3100 in combination with anti‐PD‐L1 and anti‐CTLA‐4 therapy enhanced T cell accumulation and tumor regression (Figure 5C(ii)).^[^ ^104^ ^]^ In another approach, Sun et al. engineered CAR T cells to express CXCR4, enabling chemotactic migration toward CXCL12‐enriched tumor sites. These CXCR4‐expressing CAR T cells exhibited improved tumor homing and enhanced clearance of tumor cells, demonstrating the value of chemokine receptor engineering in overcoming physical and immunological barriers. Similarly, Zhang et al. explored pharmacological inhibition of the CXCL12/CXCR4 axis as a means to mitigate MDSC recruitment and immune evasion.^[^ ^105^ ^]^

Evidently, these studies demonstrate the multifaceted role of the tumor vasculature, not just as a physical barrier but also as a dynamic immunomodulatory component of the TME. Whether through normalization, disruption, or chemokine axis targeting, modulating the tumor vasculature represents a powerful adjunct to CTT, with the potential to transform treatment outcomes in solid tumors.

#### 3.3.4 Hypoxia and Acidic TME

The TME is frequently characterized by hypoxia and acidity, consequences of rapid tumor proliferation and aberrant vasculature. These hostile physicochemical conditions profoundly impair CTT by limiting T cell infiltration, persistence, and cytotoxic activity. Overcoming the immunosuppressive effects of hypoxia and low pH is therefore essential to unlock the full therapeutic potential of CTT in solid tumors.

Stimuli‐responsive NDDS offer a promising solution by enabling precise, localized modulation of the TME. These “intelligent” nanocarriers are engineered to respond to internal tumor‐specific stimuli such as acidic pH, elevated ROS, or redox gradients, thus facilitating the controlled release of therapeutic agents within the diseased tissue.^[^ ^106^ ^]^ While the application of NDDS in immunotherapy has gained traction, their integration into CAR T cell strategies remains relatively underexplored and poorly understood.^[^ ^107^, ^108^, ^109^, ^110^ ^]^

Notable progress has been made through the development of innovative platforms. Yang et al. reported a biodegradable, hollow manganese oxide (H–MnO_2_) nanoplatform functionalized with PEG, which exhibited pH‐responsive behavior (Figure 5D(i)).^[^ ^111^ ^]^ In the acidic TME, these pH‐sensitive nanoplatforms released the payload and triggered the decomposition of tumor‐derived hydrogen peroxide to generate oxygen, thereby mitigating hypoxia and enhancing the expansion and persistence of CAR T cells.^[^ ^111^ ^]^ In another approach, Luo et al. developed an injectable immune‐microchip (i‐G/MC) system composed of alginate microspheres encapsulating IL‐15 and marine‐derived extracellular hemoglobin as an oxygen carrier.^[^ ^112^ ^]^ This system supported sustained oxygen release to remodel the hypoxic TME towards normoxic conditions and consequently enhanced TIL infiltration and activation.

In parallel, efforts to counteract TME acidity have focused on buffering agents delivered via nanoparticles. Bicarbonate‐ and calcium carbonate (CaCO_3_)‐based nanoplatforms can neutralize acidic pH, reduce lactate accumulation, and improve the metabolic landscape for effector T cells, enhancing the efficacy of monotherapies or combination therapies. Dong et al. (2023) developed calcium carbonate nanoparticle‐assembled colloidosomes (CaP CSs) that effectively reconditioned the TME by reducing acidity and hypoxia while suppressing lactate production (Figure 5(ii)).^[^ ^113^, ^114^ ^]^ In a human triple‐negative breast cancer xenograft model, these CaP CSs significantly enhanced the efficacy of antiepidermal growth factor receptor (EGFR)‐CAR T cells, boosting TIL infiltration and effector cytokine production.

Collectively, these advances highlight the potential of stimuli‐responsive NDDS to strategically reprogram the hostile TME, i.e., ameliorating hypoxia and acidity, to create a more permissive landscape for CAR T cell function. As the field moves forward, integrating smart nanomaterials with CTT holds tremendous promise for expanding the therapeutic reach of cellular immunotherapy in solid tumors.

### 3.4 Enhancing CAR T Cell Expansion and Activation

As previously noted, a major obstacle to the success of CTT in solid tumors is the onset of T cell exhaustion within the immunosuppressive TME. This dysfunctional state, driven by persistent antigen stimulation, engagement of inhibitory immune checkpoints, and metabolically hostile conditions, undermines CAR T cell efficacy, leading to reduced cytokine secretion, impaired proliferative capacity, and diminished cytolytic activity. Overcoming T cell exhaustion is therefore essential to unleashing the full therapeutic potential of CAR T cells. Toward this goal, several strategies have emerged, including optimization of in vitro and in vivo T cell expansion protocols, supplementation with proinflammatory cytokines, and the integration of immune checkpoint blockade. These combinatorial approaches hold the key to reinvigorating exhausted CAR T cells, enhancing tumor clearance, and improving clinical outcomes for patients with otherwise treatment‐resistant malignancies.

#### 3.4.1 Harnessing Immunostimulatory Cytokines for Enhanced CAR T Cell Function

Cytokines are essential modulators of immune responses to pathogens and tumors and play a critical role in maintaining lymphoid homeostasis.^[^ ^115^ ^]^ During the early phases following CAR T cell infusion, cytokines such as IL‐2, IL‐7, and IL‐15 are indispensable for supporting the expansion and persistence of therapeutic T cells. These cytokines not only enhance the in vivo durability of CAR T cells but also potentiate their cytotoxic capacity against tumor cells. However, systemic administration of cytokines carries a high risk of toxicity, prompting the development of nanotechnology‐enabled platforms to achieve localized cytokine delivery within the TME. Strategies such as nanoparticle carriers and injectable NGs and hydrogel have been engineered to provide controlled, sustained, and spatially confined cytokine release. By establishing localized immune niches, these delivery systems enhance CAR T cell survival and function within immunosuppressive TMEs, representing a promising avenue to improve the therapeutic performance of CAR T cells in solid tumors.

##### Nanoparticle Backpack

**Figure 6:**
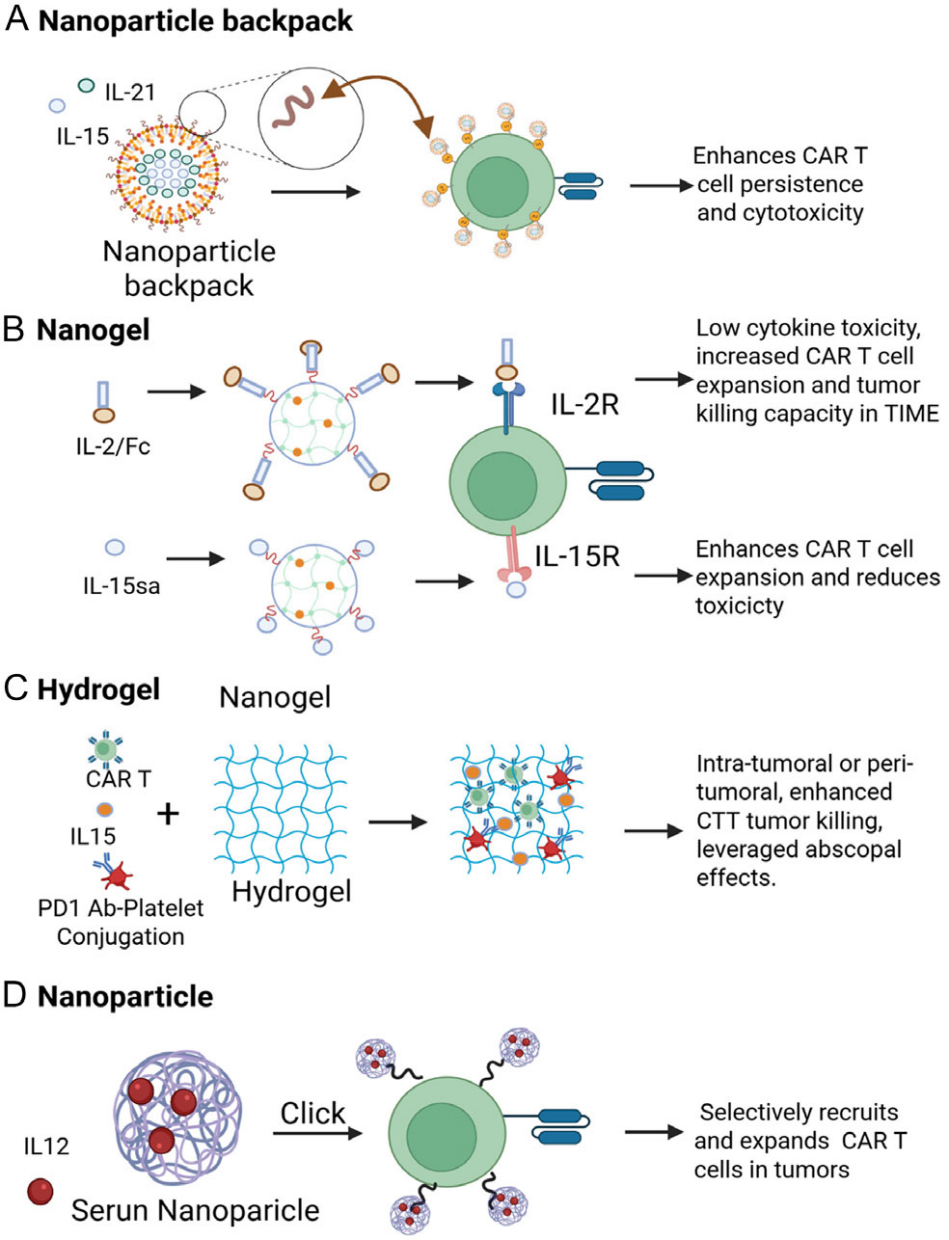
Nanotechnology‐enabled strategies to expand and activate CAR T cells. A) Nanoparticle “backpacks” were attached to CAR T cells to deliver IL‐21 and IL‐15, promoting their in vivo persistence and activation. B) A NG system was developed to codeliver IL‐12‐Fc fusion proteins and IL‐15 super‐agonists, enhancing CAR T cell expansion and tumor‐killing potential. C) A hypoxia‐responsive scaffold was used to codeliver CAR T cells, IL‐15 cytokines, and PD‐L1 antibody–platelet conjugates, supporting localized activation within the TME. D) Click chemistry was used to conjugate IL‐12‐loaded nanoparticles directly to CAR T cells, leading to enhanced expansion and function of the modified T cells. Created with BioRender.com.

To sustain T cell function within the immunosuppressive TME while avoiding systemic cytokine toxicity, Stephan et al. developed a “nanoparticle backpack” system that exploited the abundant free thiol groups on the T cell surface (**Figure** **6**A).^[^ ^50^ ^]^ This platform utilized 300‐nm multilayer lipid nanoparticles functionalized with thiol‐reactive maleimide head groups, enabling covalent attachment to T cell surface. These membrane‐anchored nanoparticles encapsulated immunomodulatory cytokines IL‐15 and IL‐21 and were engineered for controlled, antigen‐responsive release. Upon antigen recognition, the cytokines were locally released, providing continuous pseudo‐autocrine stimulation to enhance T cell expansion, persistence, and effector function. This cell‐bound delivery system eliminated the need for systemic cytokine administration, thereby reducing off‐target toxicity. In preclinical models, this nanoparticle backpack markedly improved the therapeutic efficacy of CTT against metastatic tumors, highlighting the promise of nanomaterial‐based strategies to augment T cell performance in cancer immunotherapy.^[^ ^50^ ^]^

##### Nanogels

Nanogels (NGs) have emerged as a versatile platform for targeted cytokine delivery to CAR T cells, enabling localized immune stimulation while minimizing systemic toxicity. Tang et al. developed a thiol‐reactive NG system designed to deliver IL‐15 super‐agonists such as ALT‐803 to activated T cells (Figure 6B).^[^ ^116^ ^]^ By exploiting the thiol‐rich surfaces on CAR T cells, NGs enabled localized, activation‐triggered cytokine release. This strategy led to a dramatic expansion of CAR T cells and other immune populations within the tumor and draining LNs, achieving a 16‐fold increase in intratumoral CAR T cell accumulation compared to conventional cytokine‐free cells, and an impressive 1000‐fold increase relative to cells without cytokine support.^201^ Building on this concept, Wang et al. incorporated IL‐15 delivery into an injectable hydrogel (IHG) system to support the expansion and persistence of GD2‐targeting CAR T cells.^[^ ^117^ ^]^ This approach demonstrated superior efficacy in solid tumor models, including retinoblastoma, and outperformed standard CD19‐directed CAR T therapies by enhancing T cell survival and antitumor activity. In parallel, Xie et al. developed a redox‐responsive NG formulation capable of delivering IL‐2/Fc directly to the surface of tumor‐reactive T cells, including CAR and TCR‐engineered variants.^340^ This targeted delivery achieved an 80‐fold expansion of tumor‐specific T cells compared to systemic IL‐2/Fc administration, while avoiding off‐target stimulation of tumor‐infiltrating Tregs.^[^ ^118^ ^]^ Sustained IL‐2/Fc release from the NGs promoted the differentiation of effector memory T cells and mitigated peripheral T cell deletion and anergy critical for durable responses in solid tumor immunotherapy.

##### Hydrogel

In addition to NG, bulk hydrogels have approved to be a promising platform for the localized delivery of CAR T cells and immunomodulators, offering spatial and temporal control over therapeutic release while reprogramming the TME. Hu et al. developed a multifunctional hydrogel system composed of HA, designed for direct implantation at tumor sites to codeliver CAR T cells, IL‐15 encapsulated in PNPs, and anti‐PD‐1‐conjugated platelets (Figure 6C).^[^ ^4^ ^]^ This hydrogel acted as a local immunological reservoir, enhancing CAR T cell localization, persistence, and activation within the TME. The inclusion of anti‐PD‐1 platelets leveraged tumor‐associated signals to release platelet‐derived microparticles (P‐aPD‐1), thereby reinvigorating exhausted T cells. This combinatorial strategy not only suppressed local and distant tumor recurrence but also invoked a systemic immune response, consistent with an abscopal effect, amplifying antitumor immunity beyond the primary site.^[^ ^4^ ^]^ Building upon this approach, Grosskop et al. developed a shear‐thinning, IHG composed of hydrophobically modified hydroxypropyl methylcellulose (HPMC‐C12) and RGD‐modified nanoparticles.^[^ ^119^ ^]^ This transient hydrogel was engineered to enhance CAR T cell motility and viability while supporting sustained local release of IL‐15, a key cytokine for T cell survival and expansion. In preclinical models, the hydrogel maintained over 80% of its IL‐15 payload after 7 days, significantly boosting CAR T cell persistence and cytotoxic function in the TME. These studies highlight the utility of hydrogel‐based systems in improving CAR T cell engraftment, immune activation, and therapeutic durability, especially in solid tumors where physical and immunological barriers hinder efficacy.^[^ ^119^ ^]^

##### Serum Albumin Nanoparticles

Luo et al. developed an innovative cytokine delivery system utilizing human serum albumin (HSA) nanoparticles loaded with IL‐12 to enhance the therapeutic efficacy of CAR T cell therapy (Figure 6D).^[^ ^120^ ^]^ These nanoparticles were fabricated by remodeling HSA intermolecular disulfide bond to encapsulate IL‐12 and were subsequently functionalized with dibenzocyclooctyne (DBCO) moieties. CAR T cells were bioorthogonally labeled using azide chemistry, enabling the covalent attachment of IL‐12‐loaded nanoparticles to the T cell surface via DBCO‐azide click reactions. This smart delivery system was designed to release IL‐12 in response to tumor antigen engagement, which was triggered upon an increase in thiol (mercaptan) groups on the surface of activated CAR T cells. Local IL‐12 releases promoted robust chemokine secretion and selectively recruited and amplified CD8^+^ CAR T cells within the TME. The strategy significantly enhanced intratumoral CAR T cell expansion and effector function, while minimizing the systemic toxicity typically associated with exogenous IL‐12 administration. Collectively, this approach highlights the potential of bioresponsive, nanoparticle‐mediated cytokine delivery systems to fine‐tune CAR T cell responses and improve antitumor efficacy in solid tumors.

#### 3.4.2 Engineering Lymphoid Microenvironments to Prime and Sustain CAR T Cell Responses

Tumor‐draining LNs (TdLNs) are critical immunological hubs where APCs engage with T cells to initiate adaptive immunity. Harnessing this microenvironment to enhance CTT offers a promising strategy, especially in the context of solid tumors, where immune suppression and poor T cell priming remain major obstacles. A pioneering strategy by Irvine and colleagues introduced the concept of “albumin hitchhiking,” exploiting amphiphilic molecules that bind endogenous serum albumin and are trafficking to LNs. These amphiphiles insert into APC membranes and enhance T cell activation within TdLNs.^[^ ^121^ ^]^ Building on this, Ma et al. developed amphiphile CAR T cell ligands (amph‐ligands) that piggyback on albumin to LNs, where they transfer to APC membranes and present CAR ligands. This “booster vaccine” approach enhanced CAR T cell expansion and polyfunctionality across immunocompetent tumor models—importantly, without requiring lymphodepletion or systemic cytokine support.^[^ ^122^ ^]^

Nanoparticles further extend these strategies by serving as precision vehicles for LN targeting. Nanoparticles between 10 and 100 nm efficiently drain to lymphatics, whereas larger particles (>100 nm) tend to accumulate in off‐target organs like the liver or spleen.^[^ ^123^, ^124^ ^]^ To overcome this limitation, a promising strategy is to functionalize nanoparticles to selectively target APCs within TdLNs. For instance, functionalizing liposome particles with targeting moieties, such as ganglioside GM3, a ligand for CD169 (Siglec‐1, a sialoadhesin receptor), abundantly expressed on macrophages and dendritic cells in sentinel LNs, improved delivery to macrophages and dendritic cells in TdLNs. These GM3‐liposomes promote antigen retention in endolysosomes, enhancing antigen processing and CD8^+^ T cell priming for immune synapse formation and CAR T cell activation.^[^ ^125^, ^126^ ^]^ Adjustable physicochemical properties, including size, elasticity, and surface charge, enable fine‐tuning of NP trafficking and immunomodulatory potency.

In parallel, incorporation of Toll‐like receptor (TLR) agonists into nanovaccines has demonstrated to be effective in activating innate immunity and prime T cells within TdLNs. For example, Jeanbart et al. used nanoparticles carrying TAAs and CpG oligodeoxynucleotides (TLR‐9 agonist) to significantly boost CD8^+^ T cell priming within TdLNs.^[^ ^127^ ^]^ Similarly, Lynn et al. designed TLR‐7 agonist‐conjugated micelles (~20 nm) that achieved high dendritic cell uptake (80%) and robust cytotoxic CD8^+^ T cell activation after LN trafficking.^[^ ^128^ ^]^

While leveraging native lymphoid organs is a powerful tool, biomaterial‐based LN mimetics offer a transformative alternative.^[^ ^129^ ^]^ These engineered lymphoid niches can spatially control CAR T cell activation, proliferation, and persistence, especially valuable when natural TdLNs are immunosuppressive or inaccessible.^[^ ^129^, ^130^ ^]^ These biomimetic platforms also enable the modular delivery of antigens, cytokines, and costimulatory signals. For instance, Liao et al. designed porous PLGA microspheres that mimic LN paracortical zones.^[^ ^130^ ^]^ These microspheres, functionalized with anti‐CD3/CD28, could encapsulate >38 000 CAR T cells mL^−1^ and persisted up to 49 days in vivo, sustaining CAR T cell clustering and proliferation at tumor sites. Despite the potential for lactic acid byproducts to impair T cell metabolism, these scaffolds enhanced therapeutic efficacy in xenograft models.

IHGs represent another promising delivery matrix, enabling local retention and stimulation of CAR T cells within tumor tissue.^[^ ^131^, ^132^, ^133^, ^134^, ^135^ ^]^ For example, Zhu et al. engineered a supramolecular IHG coloaded with CAR T cells and immunomodulators.^[^ ^136^ ^]^ In murine models, this depot enhanced expression of key effector cytokines (IFN‐γ and TNF‐α), facilitated sustained CAR T activation and prolonged tumor immunosurveillance, with superior tumor regression and antigen escape mitigation versus conventional CTT.

Multifunctional scaffolds can also codeliver innate immune stimulators to overcome tumor immunoevasion. Smith et al. incorporated mesoporous silica microparticles (MSMs) loaded with cyclic‐di‐GMP (a STING agonist) into biopolymer scaffolds with CAR T cells, enhancing intratumoral infiltration and activation. ^344^ Compared to diffusely distributed nano–T cell engagers (e.g., CD33‐CD3 liposomes for acute myeloid leukemia^[^ ^137^, ^138^ ^]^), such platforms offer superior spatial control and minimized systemic toxicity.

In another landmark, Agarwalla et al. developed the MASTER (Multifunctional Alginate Scaffold for T cell Engineering and Release) platform, an implantable 3D alginate matrix that supports CAR T expansion and functional programming.^[^ ^139^ ^]^ MASTER significantly enhanced CAR T persistence and cytolytic activity in murine tumor models.

In summary, both LN‐targeted nanodelivery systems and engineered lymphoid scaffolds exemplify the next generation of spatially programmed immunotherapies. By emulating or augmenting lymphoid environments, these approaches optimize CAR T cell priming, persistence, and therapeutic performance, laying the groundwork for safer, more effective CTT strategies in solid tumors. Moving forward, optimization of scaffold biodegradability, immunomodulatory payloads, and integration with smart drug delivery systems will be critical to enhancing clinical translation and overcoming barriers in solid tumor CTT.

#### 3.4.3 Engineered Artificial APCs (aAPC) for Potentiating CAR T Cell Activation

**Figure 7:**
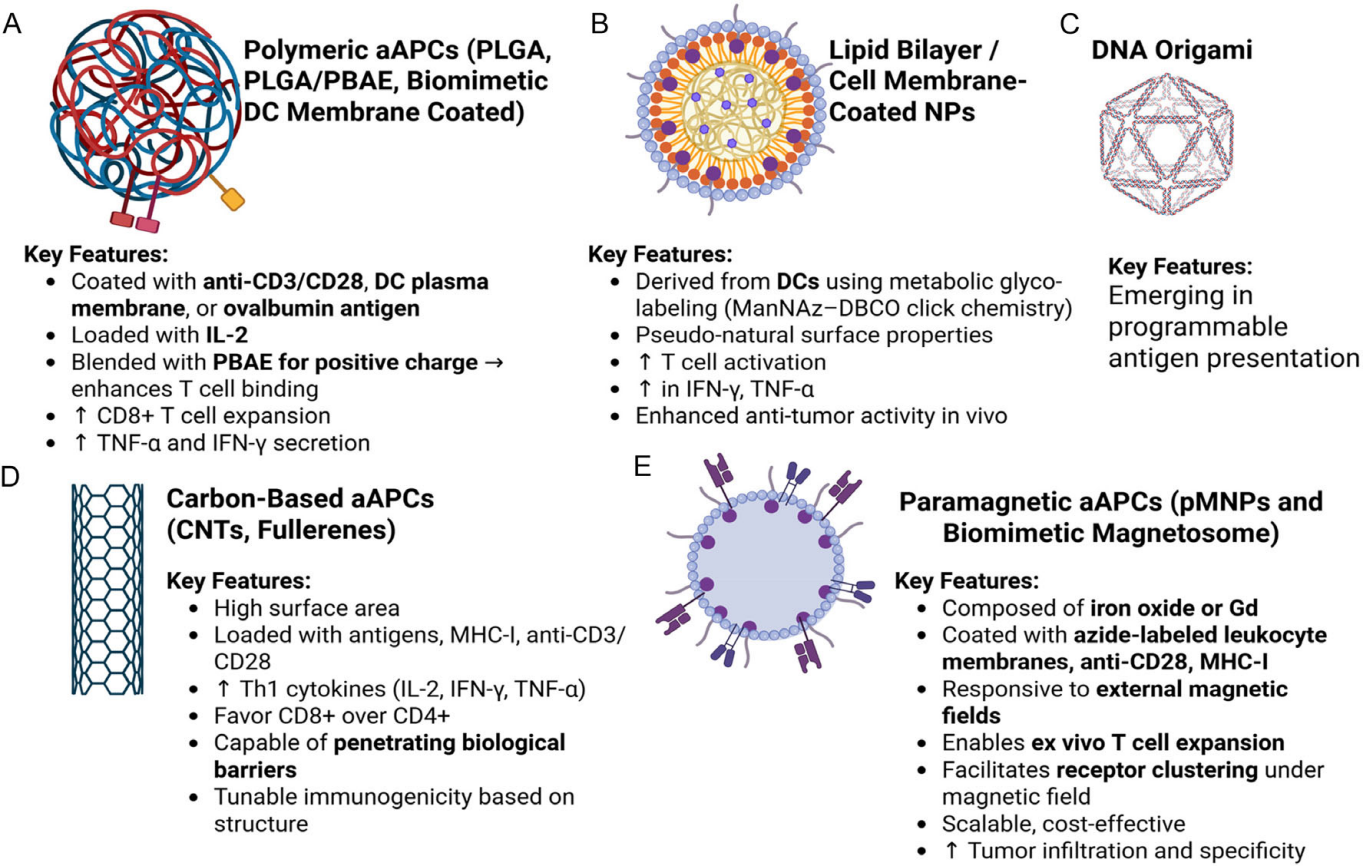
aAPC platforms for enhancing CAR T cell activation and expansion. A) Polymeric aAPCs—synthetic polymer particles functionalized with stimulatory ligands such as anti‐CD3 and anti‐CD28 antibodies simulate the primary and costimulatory signals required for robust CAR T cell activation and expansion in vitro. B) Liposome‐based aAPCs—lipid vesicles engineered to present membrane‐bound cytokines (e.g., IL‐15) and costimulatory molecules provide a biocompatible platform for enhancing CAR T cell proliferation, survival, and effector function. C) DNA origami‐based aAPCs—these nanoscale scaffolds utilize precisely folded DNA structures to spatially organize signaling ligands with nanometer resolution, allowing for fine‐tuned and programmable presentation of activation cues to CAR T cells. D) Carbon‐based aAPCs—CNTs, graphene, or other carbon nanostructures offer high surface area and functional versatility for immobilizing ligands and cytokines, supporting multivalent T cell engagement and signal delivery. E) Paramagnetic aAPCs and biomimetic magnetosomes—magnetic nanoparticle‐based aAPCs can be manipulated using external magnetic fields to cluster and activate CAR T cells more efficiently, while magnetosome‐inspired structures enhance targeting and spatiotemporal control of T cell stimulation. Created with BioRender.com.

Nanoparticle‐based aAPCs represent a transformative approach to enhance CAR T cell activation and streamline ACT (**Figure** **7**).^[^ ^84^ ^]^ These engineered aAPCs recapitulate key immunological cues of natural APCs namely antigen presentation, costimulatory signaling, and cytokine delivery using diverse nanomaterials such as PNPs, carbon nanostructures, magnetic nanoparticles, liposome, and DNA origami platforms.

A representative strategy by Xiao et al. utilized PNPs cloaked with dendritic cell‐derived plasma membranes via metabolic glyco‐labeling.^[^ ^140^ ^]^ Azide‐functionalized N‐acetylmannosamine (ManNAz) enabled surface labeling of dendritic cells, which subsequently bound DBCO‐functionalized PNPs to form membrane‐coated aAPCs. This platform boosted T cell activation in vitro by 26%, significantly increased IFN‐γ and TNF‐α secretion and improved antitumor efficacy in vivo. Early efforts using PLGA‐based aAPCs, functionalized with anti‐CD3 and anti‐CD28 antibodies, demonstrated nonspecific but potent CD8^+^ T cell expansion.^[^ ^140^ ^]^ These nanoparticles effectively integrated antigen stimulation with IL‐2 release, which outperformed soluble IL‐2 by upregulating CD25 expression on both CD4^+^ and CD8^+^ T cells, thereby enhancing T cell activation and proliferation. (Figure 7A)^[^ ^141^ ^]^ However, the negatively charged PLGA‐based aAPCs limited their biodistribution by preferentially binding scavenger receptors in the spleen or liver. To overcome this, Rhodes et al. developed hybrid aAPCs using PLGA and positively charged poly(β‐amino ester) (PBAE).^[^ ^142^ ^]^ This dual‐polymer design yielded a 1.5‐fold increase in ligand density, a 35‐fold enhancement in T cell binding (measured via MFI), and a 5‐fold expansion in CD8^+^ T cells, all attributed to optimized electrostatic interactions and improved antigen‐specific T cell engagement.

Carbon nanomaterials offer additional advantages for aAPC design due to their unique physicochemical properties. Carbon nanotubes (CNTs), with high surface area and cellular penetrability, can present antigens and stimulatory ligands to promote robust T cell proliferation. (Figure 7D)^[^ ^84^ ^]^ Although not commonly used, carbon nanoparticles with all three dimensions in nanometer range could bypass biological barriers and reach areas that conventional drugs cannot, making them highly valuable in drug delivery.^[^ ^143^ ^]^ For example, Liu et al. utilized the carbon allotrope C_60_(OH)_20_ (a water‐soluble fullerene) to augment CD8^+^ T cell responses and bias cytokine secretion toward a Th1 profile (IL‐2, IFN‐γ, TNF‐α), reducing Th2‐associated immunosuppression. Similarly, multi‐walled (CNTs (MWCNTs) have shown promise for antigen delivery and T cell priming.^[^ ^144^, ^145^ ^]^ However, variability in formulation and potential biocompatibility concerns necessitate rigorous evaluation prior to clinical translation.^[^ ^146^ ^]^

Paramagnetic nanoparticles (pMNPs), typically composed of iron oxide or gadolinium, allow magnetic guidance and clustering of TCRs to enhance T cell activation, selection, and expansion (Figure 7E).^[^ ^147^ ^]^ Bieler et al. demonstrated that pMNPs could support the expansion of tumor‐specific T cells ex vivo in both murine and human models. More recently, Zhang et al. engineered biomimetic magnetosomes by assembling magnetic nanoclusters coated with azide‐labeled leukocyte membranes and conjugating key T cell activating ligands (e.g., anti‐CD28, MHC‐I complexes).^[^ ^148^ ^]^ These magnetosomes promoted scalable, antigen‐specific CAR T cell manufacturing with improved functional outcomes and safety profiles.^[^ ^148^, ^149^ ^]^ Their design supports immune synapse formation and mimics physiological T cell priming during CTT.

Collectively, these innovations underscore the potential of aAPCs as scalable, tunable, and potent alternatives to natural APCs. Continued refinement of aAPC physicochemical properties such as size, surface charge, ligand density, and biodegradability will be essential to maximize therapeutic impact and facilitate widespread adoption in cellular immunotherapy.

### 3.5 Synergizing Nanomedicine and CAR CTT: Engineering the TME for Immunological Remodeling

Beyond serving as delivery vehicles or scaffolds, nanomedicine‐based therapies offer transformative potential to modulate the immune response within the TME, particularly by reprogramming immunosuppressive elements that hinder effective antitumor immunity. One prominent mechanism is the induction of immunogenic cell death (ICD), a form of cell death that promotes the release of damage‐associated molecular patterns (DAMPs) and TAAs. These molecules are crucial for dendritic cell activation and subsequent cytotoxic T lymphocyte (CTL) priming, ultimately enhancing CAR T cell activity.^[^ ^150^ ^]^ Local ablative therapies such as chemotherapy, radiotherapy (RT), photothermal therapy (PTT), photodynamic therapy (PDT), and sonodynamic therapy (SDT) have demonstrated the capacity to induce ICD and reshape the TME to support cellular immunotherapies.^[^ ^151^, ^152^, ^153^ ^]^ Together, PTT, SDT, and PDT highlight the potential of nanomedicine‐based local ablative therapies to induce ICD, enhance antigen presentation, improve immune cell infiltration, and reshape the TME. These strategies create a synergistic framework for potentiating CAR T cell therapies in solid tumors, bridging the gap between immune engineering and tumor immunomodulation.

#### 3.5.1 PTT

PTT represents a minimally invasive and nontoxic approach to remodel the TME and overcome physical and immunological barriers limiting CAR T cell infiltration and persistence. Upon exposure to near‐infrared (NIR) light, PTT induces localized hyperthermia that disrupts the dense ECM, promotes vasodilation, and increases vascular permeability—key factors that facilitate T cell trafficking into solid tumors. Studies have shown that PTT can improve the expression and surface presentation of CAR molecules, further directing engineered T cells toward tumor‐specific antigens.^[^ ^154^, ^155^ ^]^ Moreover, NIR‐triggered PTT, when precisely tuned in dose and localization, can induce necrosis and programmed cell death (PCD) while maintaining antigen integrity, creating a highly immunogenic TME that supports CAR T cell expansion and persistence.^[^ ^156^ ^]^ Various nanoplatforms have been developed to enhance PTT including metal‐based (e.g., gold, Cu_2_‐xS), carbon‐based (e.g., graphene, black phosphorus (BP)), polymeric, or lipid‐based systems. For instance, Hu et al. engineered NIR‐responsive indocyanine green (ICG)‐loaded nanoparticles (NP‐ICG), which induced local hyperthermia but also augmented antigen release and proinflammatory cytokine secretion, enhancing CAR T cell trafficking and functionality.^[^ ^154^ ^]^ Chen et al. advanced this concept by designing nanophotosensitizer‐conjugated CAR T cells (CT‐INPs) that, upon NIR irradiation, penetrated deeper into tumors and significantly suppressed tumor growth while elevating IL‐2 and IFN‐γ as a result of mild hyperthermia.^[^ ^157^ ^]^ Liang et al. develop BP‐based erythrocyte‐membrane‐coated platforms systems that triggered ROS generation and tumor antigen release post‐NIR, promoting CAR T cell accumulation in tumors.^[^ ^158^ ^]^ Similarly, Zhu et al. used HA@Cu_2_‐xS‐PEG nanoenzymes, harnessing photothermal–nanocatalytic (PNC) synergy to modulate the immunosuppressive TME and promote CAR T cell activation via TSA release.^[^ ^159^ ^]^ In another innovative strategy, Shi et al. created a multifunctional theragnostic nanoparticle (FA‐Gd‐GERTs@Ibrutinib) combining folate receptor targeting, gadolinium‐based imaging agents, and Raman‐enhanced PTT.^[^ ^160^ ^]^ This platform facilitated enhanced accumulation of CAR T cells and synergistically improved tumor ablation and immune activation in murine models. Together, these approaches highlight the potential of PTT to convert immunosuppressive tumors into CAR T‐permissive environments.

#### 3.5.2 SDT

SDT utilizes noninvasive ultrasound (US) to activate tumor‐localized sonosensitizers, leading to ROS production that directly induce tumor cell apoptosis and ICD. This ROS‐mediated tumor lysis results in epitope spreading, which facilitates DC maturation and subsequent activation of CTLs, thereby potentiating a systemic antitumor immune response in synergy with CTT. Despite its promise, clinical translation of SDT remains limited by heterogeneous tumor responses and nonoptimized sonication parameters, necessitating precision‐targeted strategies to improve its therapeutic index. To enhance precision, focused US (FUS) has been used to achieve spatiotemporally controlled hyperthermia and increase vascular permeability at tumor sites for improved immune cell infiltration, thereby amplifying both the direct cytotoxic and immunomodulatory effects of SDT.^[^ ^161^ ^]^ Nanocarriers have been used to selectively accumulate sonosensitizer in the TME, minimizing off‐target toxicity and maximizing ROS production upon US exposure. Wu et al. introduced a novel FUS‐responsive CAR‐T cell platform that uses a heat‐shock protein (HSP)‐driven promoter cassette for spatially restricted CAR expression.^[^ ^162^ ^]^ This system enables reversible, localized activation of CAR‐T cells within tumors, allowing for high therapeutic precision while mitigating systemic toxicity. In another attempt, Wang et al. introduced fluorine‐rich oscillatory mesoporous organosilica nanoparticles (FRMONs) loaded with ICG.^[^ ^163^ ^]^ Upon US activation, they produced ROS through the ICG‐mediated sonodynamic process. The elevated ROS level led to tumor cell ablation and induced ICD (marked by elevated calreticulin (CRT) levels and high mobility group box 1 (HMGB1)). These DAMPs facilitated DC maturation and antigen cross presentation in tdLNs, ultimately promoting systemic CD8^+^ T cell (CTL) activation, characterized by elevated IFN‐γ production. Clearly, these studies highlight the potential of SDT to transform the TME from an immunosuppressive niche into a proinflammatory milieu, conducive to CAR T cell trafficking and activation.

#### 3.5.3 PDT

PDT combines photosensitizers (PSs), light, and oxygen to generate cytotoxic ROS that trigger ICD. To improve PS stability, pharmacokinetics, and targeted accumulation in tumor, nanocarriers such as liposomes, PNPs, gold nanostructures, metal‐organic frameworks, and other nanoparticles are frequently used to encapsulate PSs.^[^ ^164^ ^]^ Upon light activation, PDT‐induced ROS not only mediate tumor cell lysis but also promote the release of DAMPs and TAAs, promoting DC maturation and subsequent T cell priming. Recent studies demonstrate that combining PDT‐induced immunogenic modulation with CAR‐T therapy enhances T cell infiltration, activation, and cytotoxicity.^[^ ^165^ ^]^ This integrative approach transforms PDT from a primarily localized cytotoxic modality into a potent immunostimulatory strategy, capable of initiating tumor antigen release, DC activation, and epitope spreading. By promoting the recruitment and activation of endogenous T cells, these nanocarrier‐enabled PDT systems reprogram the TME and convert immunologically “cold” tumors into “hot” immunogenic niches.^[^ ^166^ ^]^ This antigen‐driven immune priming synergizes with CAR T cell therapy, fostering enhanced expansion, persistence, and cytotoxicity of engineered T cells within otherwise resistant tumor niches. Consequently, multifunctional nanocarrier‐enabled PDT offers a next‐generation platform for augmenting the efficacy of CTT, especially in solid tumors.

Liposomal and MOF‐based nanoparticles have been used to encapsulate hydrophobic PSs, improving their solubility, pharmacokinetics, and tumor accumulation. Upon light activation, these systems generate ROS, induce ICD, and facilitate dendritic cell maturation and T cell priming.^[^ ^167^ ^]^ Gold nanoframeworks and PNPs loaded with PSs have been developed for targeted tumor delivery. These platforms enhance ROS generation, promote the release of DAMPs and TAAs, and convert immunologically “cold” tumors into “hot” ones, thereby supporting T cell infiltration and activation.^[^ ^164^ ^]^ Recent studies demonstrate that combining nanoparticle‐mediated PDT with CAR T cell therapy enhances T cell infiltration, activation, and cytotoxicity within solid tumors, overcoming immunosuppressive barriers and improving therapeutic outcomes.^[^ ^168^ ^]^

### 3.6 Nanotechnology‐Enabled Strategies for Enhancing the Safety and Controllability of CTT

Despite the groundbreaking success of CTT in hematologic malignancies, its clinical application, especially in solid tumors, remains constrained by severe toxicities, including CRS and ICANS. These complications arise from uncontrolled CAR T cell activation and massive systemic cytokine release. Additionally, off‐tumor, on‐target effects pose significant safety risks due to the lack of spatial control over T cell activity. Nanotechnology offers powerful solutions to these challenges by enabling localized cytokine modulation, external control over CAR expression, and precise therapeutic activation, all of which support safer and more clinical implementation.

#### 3.6.1 Sequestration of Cytokines

A promising approach to mitigating CRS centers on the localized sequestration and neutralization of proinflammatory cytokines. Among them, IL‐6 plays a central role in CRS pathogenesis, particularly in solid tumor‐directed CTT. To address this, Li et al. developed an IL‐6 sponge (IL6S) composed of a thermosensitive HG functionalized with anti‐IL‐6 antibodies.^[^ ^169^ ^]^ This injectable smart biomaterial forms a subcutaneous depot capable of sustained cytokine capture in vivo. In both immunodeficient and humanized mouse models, IL6S significantly reduced circulating levels of IL‐6, CCL2, TNF‐α, G‐CSF, IL‐3, and IL‐10, effectively alleviating key CRS symptoms such as fever, hypotension and weight loss, while preserving CAR T cell antitumor activity. Compared to free antibody administration, IL6S provided prolonged, tunable cytokine control and improved survival outcomes. Importantly, the HG could be safely and reversibly removed via syringe aspiration upon cooling, making it a clinically tractable and modular safety tool for CAR T cell immunotherapy.^[^ ^169^ ^]^ In addition to IL6S, other nanomaterials such as neutrophil‐inspired “cytokine sponges” and nanodiamonds have also shown strong potential potent cytokine adsorption capabilities.^[^ ^170^, ^171^ ^]^ These platforms offer complementary, broad‐spectrum strategies for mitigating CRS‐related toxicities by targeting multiple proinflammatory cytokines while preserving immune function.

#### 3.6.2 Engineering Light‐Sensitive CAR T Cells

To further improve safety, particularly with respect to off‐tumor toxicity, external control of CAR T cell activation via optogenetic and photothermal mechanisms has gained a great attention. These systems allow for spatiotemporal restriction of T cell activity to tumor beds, minimizing systemic exposure and immune‐related adverse events.^[^ ^172^ ^]^ Miller et al. pioneered a platform where CAR T cells were conjugated with gold nanorods (GNRs) that respond to NIR‐I light.^[^ ^173^ ^]^ Upon localized irradiation, GNRs generated mild hyperthermia in the TME, triggering site‐specific release of immunomodulatory cytokines such as IL‐15, thereby enhancing antitumor immunity with minimal systemic cytokine surge and toxicity. Nguyen et al. advanced this concept using upconversion nanoplates (UCNPs), which converted deep tissue‐penetrating NIR‐II light into blue light.^[^ ^174^ ^]^ The light activates light‐inducible CAR expression systems (LiCARs), allowing for deep‐tissue CAR T cell activation with high spatiotemporal resolution. In murine models, this platform achieved tumor‐restricted CAR expression, improved tumor control, and significantly reduced off‐target toxicities. These nanotechnology‐based light‐gated control systems offer clinicians real‐time command over CAR T cell behavior, functioning as precision safety switches that could be integrated into next‐generation immunotherapies.

By integrating nanomedicine with immunoengineering, innovative strategies such as cytokine sponges and light‐responsive CAR systems provide critical advancements in CAR T cell safety management. These platforms offer clinician‐guided control, reduce systemic toxicity, and improve therapeutic precision, which collectively pave the way for broader and safer application of CAR T cell therapies in solid tumors and other high‐risk malignancies.

## 4 Conclusion and Perspectives

The convergence of CAR‐T cell therapies and nanomedicine has emerged as a transformative strategy in the advancement of precision immunotherapy, particularly in overcoming the limitations of conventional CTT in solid tumors. Preclinical studies have consistently demonstrated that integrating nanotechnology with CAR‐T platforms can improve efficacy, enhance tumor targeting, and mitigate toxicities—hallmarks critical for broader clinical success. By exploiting the unique physicochemical properties of nanoscale materials, such as nanoparticles, liposomes, and nanobodies, researchers have developed delivery systems that not only improve CAR‐T cell homing and expansion within the TME but also facilitate codelivery of immunomodulators that reshape the TME to support CAR‐T cell function. Nanomedicine enables the protection and sustained release of therapeutic payloads, improving their stability, pharmacokinetics, and bioavailability. This precise spatiotemporal control enhances therapeutic efficacy while minimizing systemic exposure and associated adverse effects, such as CRS and off‐tumor toxicities. Moreover, the surface of nanomaterials can be engineered with targeting ligands or antibodies to selectively engage tumor antigens or immune‐suppressive components of the TME, thereby increasing CAR‐T cell specificity and therapeutic precision. These targeted strategies hold particular promise for personalized cancer immunotherapy, where patient‐specific tumor profiles guide the rational design of nanomedicine‐CAR‐T combinations.

Despite the promising advances in nanomedicine‐enabled CTT, several challenges remain unresolved, including concerns about the limited and unsustainable efficacy of current approaches, the potential carcinogenicity of viral vectors, the toxicity and long‐term clearance of nanomaterials, and the complexity, cost, and scalability of manufacturing customized nanoenabled CAR‐T systems. These limitations continue to hinder the clinical translation of CAR‐T therapy for solid tumors. To achieve broader clinical adoption, several key areas warrant further development, including (i) rational design of nanoparticles to remodel the TME through targeted delivery of small‐molecule inhibitors, siRNA, cytokine modulators, or their combinations, (ii) creation of multifunctional “theranostic” nanoparticles that integrate imaging reporters to enable real‐time monitoring of CAR‐T cell biodistribution, activation, and therapeutic response, (iii) combination of nanomedicine with conventional or immune‐stimulating modalities—such as photodynamic, radiotherapeutic, or chemotherapeutic agents—to enhance antigen release and promote CAR‐T recruitment, and (iv) development of in vivo CAR‐T generation platforms utilizing scalable, nonviral nanoparticles to deliver CAR transgenes or gene‐editing reagents directly within the host, thereby eliminating the need for costly ex vivo manufacturing. Collectively, these strategies are expected to enable the next generation of CAR‐T therapies with enhanced tumor specificity, safety, and durability, positioning nanomedicine as a critical enabler in realizing the full therapeutic potential of engineered cell therapies against solid tumors.

Besides the research endeavors, the integration of nanotechnology into CTT also demands rigorous alignment with ethical, regulatory, and manufacturing standards to ensure patient safety and equitable access. Collaborative efforts across scientific disciplines, regulatory agencies, policymakers, patient advocacy groups will be essential to navigate these hurdles and bring safe, effective nano‐CAR T therapies to the clinic.

In summary, nanomedicine offers powerful tools to amplify the therapeutic potential of CAR T cells, particularly in the context of solid tumors and immunosuppressive microenvironments. As the field progresses, next‐generation platforms combining nanotechnology, immunoengineering, and systems biology are poised to redefine cancer treatment paradigms and improve patient outcomes in a personalized, targeted, and safer manner.

## Conflict of Interest

The authors declare no conflict of interest.
